# Mechanismbased role of the intestinal microbiota in gestational diabetes mellitus: A systematic review and meta-analysis

**DOI:** 10.3389/fimmu.2022.1097853

**Published:** 2023-03-03

**Authors:** Min Yan, Xiaoying Guo, Guiyuan Ji, Rui Huang, Dongyi Huang, Zhifeng Li, Dantao Zhang, Siyi Chen, Rong Cao, Xingfen Yang, Wei Wu

**Affiliations:** ^1^School of Public Health, Southern Medical University, Guangzhou, China; ^2^Guangdong Provincial Institute of Public Health, Guangdong Provincial Center for Disease Control and Prevention, Guangzhou, China

**Keywords:** gestational diabetes mellitus, gut microbiota, meta-analysis, systematic review, insulin resistance

## Abstract

**Background:**

Metabolic disorders caused by intestinal microbial dysregulation are considered to be important causes of gestational diabetes mellitus (GDM). Increasing evidence suggests that the diversity and composition of gut microbes are altered in disease states, yet the critical microbes and mechanisms of disease regulation remain unidentified.

**Methods:**

PubMed^®^ (National Library of Medicine, Bethesda, MD, USA), Embase^®^ (Elsevier, Amsterdam, the Netherlands), the Web of Science™ (Clarivate™, Philadelphia, PA, USA), and the Cochrane Library databases were searched to identify articles published between 7 July 2012 and 7 July 2022 reporting on case–control and controlled studies that analyzed differences in enterobacteria between patients with GDM and healthy individuals. Information on the relative abundance of enterobacteria was collected for comparative diversity comparison, and enterobacterial differences were analyzed using random effects to calculate standardized mean differences at a *p*-value of 5%.

**Results:**

A total of 22 studies were included in this review, involving a total of 965 GDM patients and 1,508 healthy control participants. Alpha diversity did not differ between the participant groups, but beta diversity was significantly different. *Firmicutes*, *Bacteroidetes*, *Actinobacteria*, and *Proteobacteria* were the dominant bacteria, but there was no significant difference between the two groups. Qualitative analysis showed differences between the groups in the *Firmicutes*/*Bacteroidetes* ratio, *Blautia*, and *Collinsella*, but these differences were not statistically different.

**Conclusion:**

Enterobacterial profiles were significantly different between the GDM and non-GDM populations. Alpha diversity in patients with GDM is similar to that in healthy people, but beta diversity is significantly different. *Firmicutes*/*Bacteroidetes* ratios were significantly increased in GDM, and this, as well as changes in the abundance of species of *Blautia* and *Collinsella*, may be responsible for changes in microbiota diversity. Although the results of our meta-analysis are encouraging, more well-conducted studies are needed to clarify the role of the gut microbiome in GDM. The systematic review was registered with PROSPERO (https://www.crd.york.ac.uk/prospero/) as CRD42022357391.

## 1 Introduction

Gestational diabetes mellitus (GDM) is characterized by insufficient insulin secretion and impaired glucose intolerance during pregnancy (1). It has been estimated that over the past 20 years the worldwide prevalence of GDM has been up to 14% of pregnancies, though estimates may vary among regions depending on diagnostic criteria (2). GDM has been associated with adverse pregnancy outcomes among pregnant women including dystocia, macrosomia, neonatal hypoglycemia, and birth injuries (3). In addition, it has been reported that, in the long run, GDM is associated with type 2 diabetes mellitus (T2DM), metabolic syndrome, and cardiovascular disease, making it more potential of developing T2DM than ordinary pregnancies. It is generally believed that GDM develops when pancreatic beta cells fail to produce sufficient insulin to meet the demands of the relevant tissues for blood glucose regulation (4, 5). Therefore, GDM can be considered a manifestation of prediabetes in the form of impaired glucose tolerance in non-pregnant individuals (1). Undoubtedly, diagnosis and treatment can not only reduce the risk of perinatal complications but can also reduce the economic burden on both patients and countries. The human gut microbiota is considered to have a profound influence on host metabolism (6), and alteration of the homeostasis of the intestinal microbiota can have far-reaching consequences. For instance, a reduced number of bacteria such as bifidobacteria and *Bacteroides* affects lipid metabolism, whereas an increased number of enterobacteria can lead to insulin resistance (6–9). Although previous studies have explored different populations, most have focused on the components of and changes in the gut microbiota in GDM during different pregnancy periods. Few studies have explored differences in the microbiota in patients with GDM and healthy controls. In addition, the results have been inconsistent and findings cannot be reproduced. Specific and meticulous pathogenesis remains to be identified.

Various mechanisms explaining the link between the gut microbiota and GDM have been proposed. A lack of SCFAs (short-chain fatty acids) is one reason that has been suggested. This is particularly common among those consuming Western-style diets, which are known to be low in fiber and digestible carbohydrates, which may contribute to a reduction in microbial diversity and cause microbial dysbiosis. Such diets could also change the profile of gut microbiota, so as to impair the integrity of the wall of the intestine and cause gut permeability. Thus, one effect of insufficient SCFAs may be translocation of toxins from the gut lumen to the systemic circulation (10).

Another proposed mechanism is associated with some other foods, such as fish and red meat. The intestinal flora can produce trimethylamine *N*-oxide (TMAO) by metabolizing those meat, thus affecting the immune system. In addition, activation of the intracellular thioredoxin-interacting protein (TXNIP) leads to an in increase in the expression of the NLRP3 gene [NOD-like receptor (NLR) family pyrin domain containing 3] and increased inflammatory markers in blood, especially tumor necrosis factor-α (TNF-α), interleukin 6 (IL-6), interleukin 18 (IL-18), and interleukin-1β (IL-1β) (11–13). Dietary TMAO then further increases fasting insulin levels and homeostasis model assessment for insulin resistance (HOMA-IR) by inducing adipose tissue inflammation (14), exacerbating impaired glucose tolerance. The mechanisms affected by the gut microbiota may indicate possible treatments for diabetes mellitus.

The aim of this review was to collect evidence from cohort and case–control studies to provide a theoretical basis for the diagnosis, intervention, and treatment of diseases linked to the gut microbiota by analyzing differences in enterobacteria and exploring inflammation and possible immune mechanisms associated with disease pathophysiology.

## 2 Materials and methods

The scheme of the metareview has been registered with PROPSERO (as CRD42022357391) and searches were conducted in accordance with the updated 2008 Preferred Reporting Items for Systematic Reviews and Meta-Analyses (PRISMA) statement (15) and checklist.

### 2.1 Data sources and search strategy

A systematic literature search of four databases [PubMed^®^ (National Library of Medicine, Bethesda, MD, USA), the Cochrane Library, Embase^®^ (Elsevier, Amsterdam, the Netherlands), and Web of Science™ (Clarivate™, Philadelphia, PA, USA)] was conducted by combining medical subject headings (MeSH) words with free words. We selected articles in English published between 7 July 2012 and 7 July 2022 and focused on human studies.

The search strategy was built by combining the following MeSH words with free words: (((“Diet”[Mesh]) OR (“Life Style”[Mesh]) OR (“Exercise”[Mesh]) OR (“Motor Activity”[Mesh]) OR (“Probiotics”[Mesh]) AND ((“Cohort Studies”[Mesh]) OR (Cohort studies [Title/Abstract])) AND ((16S rRNA [Title/Abstract])) AND ((“Diabetes, Gestational”[Mesh])). Various free words were added to improve the search results and identify articles that might otherwise have been missed. The search strings are provided in **Supplementary Table 1**.

### 2.2 Eligibility criteria

Titles and abstracts were screened by two investigators. Any disagreements between the two researchers were resolved by a third one. Before the formal literature selection, the three investigators were trained to ensure that they had consistent screening standards. Studies were included if they met the following criteria (1): they were cohort studies or case studies among pregnant women with GDM (2); metagenomics sequencing or 16S rRNA sequence analysis was carried out (3); they reported maternal outcomes such as HbA1c, fasting blood glucose level, and gestational weight gain (4); they were published from 7 July 2012 to 7 July 2022 (5); they were published in English; and (6) the population studied was aged >18 years.

Articles were excluded if they met any of the following criteria (1): they reported on trials that were not carried out in humans (2); they were non-randomized controlled studies (3); they analyzed probiotics in conjunction with other GDM therapies in the same intervention group (4); they were abstracts, case reports, expert opinions, reviews, letters, or editorials; or (5) they lacked sufficient data or did not meet the inclusion criteria.

### 2.3 Data extraction and synthesis

Articles on pregnant women under 45 years old who had been diagnosed with GDM at a specific time point were chosen. The diagnosis of GDM was confirmed by a national or international standard. A further requirement was that the control groups should be healthy and the GDM group should not also have other metabolic diseases.

All articles were screened, and those deemed ineligible by the two researchers were removed; any disagreements were resolved by the third researcher. Detailed information was then recorded in an Excel spreadsheet. This included basic information such as authors, publication year, district, and study types, as well as maternal outcomes such as HbA_1c_ and fasting blood glucose levels and details of the gut microbiome, including diversities in richness, evenness, and similarity between communities.

### 2.4 Quality assessment and risk of bias

**Figure 1:**
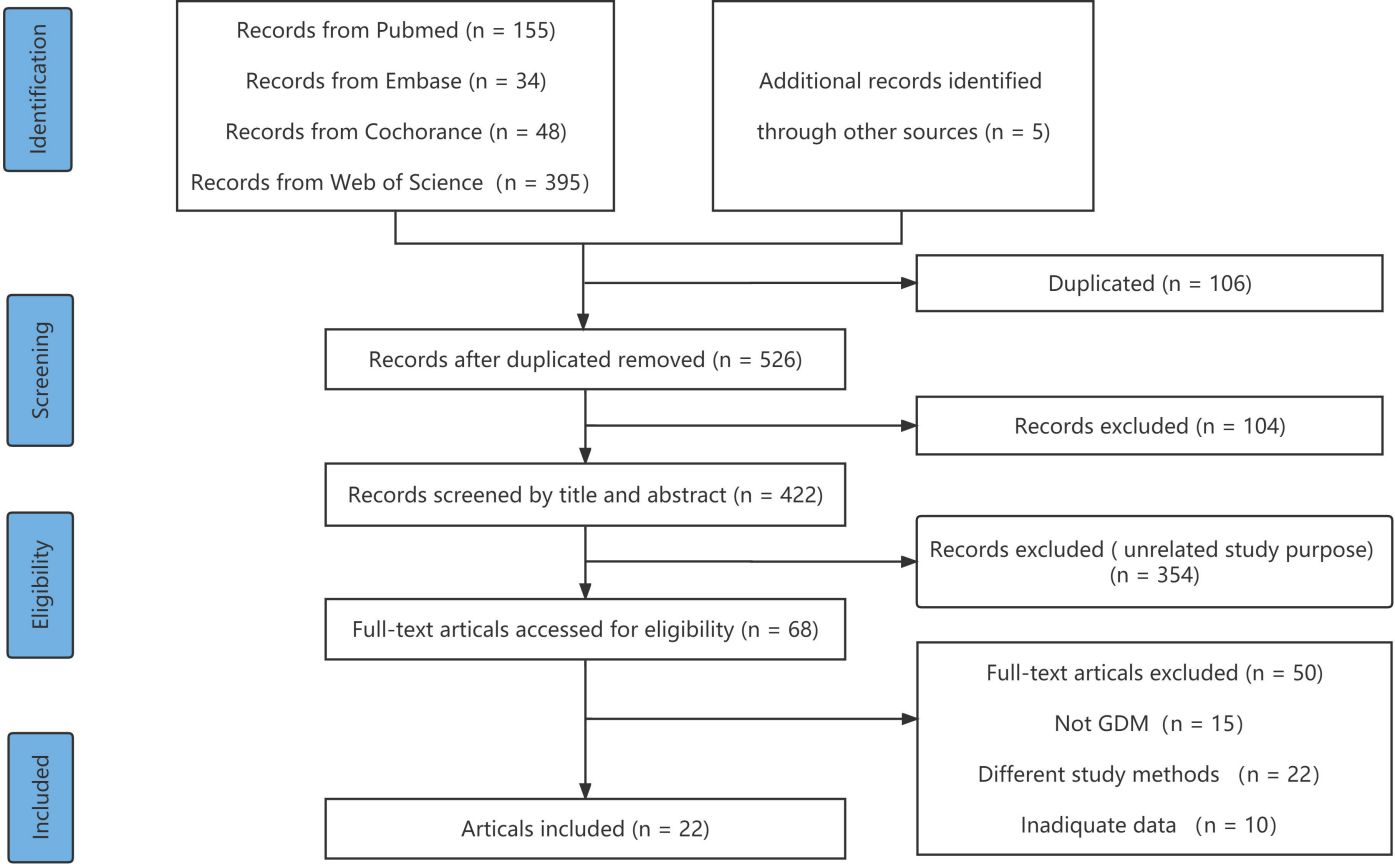
The PRISMA flow diagram of study identification.

We used the Newcastle–Ottawa Scale (NOS), which was developed for the assessment of cohort studies and case studies, to score the included studies (16). For the purpose of reaching accurate methodological quality, the NOS was evaluated through three dimensions (1): selection (2), comparability, and (3) outcome. A quality score ranging from 0 to 9 was obtained through a rating algorithm, with a score of 0–5 meaning poor quality, a score of 6–7 meaning moderate quality, and a score of 8–9 meaning high quality. The specific scores of each article according to the NOS are shown in **Figure 1** and **Supplementary Table 2**.

### 2.5 Statistical analysis

Standardized mean differences (SDMs) were used to summarize and study differences among the studies and relevant measures, and 95% confidence intervals (CI) were used to estimate the differences in gut microbiota diversity between the GDM and the non-GDM groups. Operational taxonomic unit (OTU) data for each study were analyzed using RevMan 5.3 software, provided by the Cochrane Collaboration Network. Meta-analysis was performed using a random-effects model or a fixed-effects model. Sensitivity analyses were performed using the Egger test. Forest plots were created to visualize differences in microbial community structure between samples using a random-effects (RE) model and a fixed-effects (FE) model according to the reported *I*^2^-value. An *I*^2^-value > 50% and a *p*-value > 0.05 were considered statistically significant (17).

Qualitative data, such as the relative abundance of specific genera, were recorded for analysis. Subgroup analyses for confounding factors, such as diet, were performed. Because qualitative beta diversity was not provided, descriptive analyses could not be performed. We therefore chose to perform semiquantitative analysis. If two or more articles reported consistent study results, it was considered that the results were related to the disease and were worthy of further exploration and explanation.

## 3 Results

### 3.1 Search results and study eligibility

A total of 632 articles identified from four databases (PubMed, 155; Embase, 34; the Cochrane Library, 48; and Web of Science, 395) were retrieved. Of these, 106 articles were duplicates and were removed, 354 articles were excluded because they reported on different study purposes, and 20 were excluded because they described other study designs. After excluding publications that did not meet the inclusion criteria, 22 (8, 18–27) studies remained and were subjected to full-text scanning for this systematic review. The flow chart illustrating the selection process is provided in **Figure 1**.

Among all the selected studies, 18 studies were conducted in Asia (16 from China, one from Malaysia, and one from Japan). Nine were cohort studies, while 13 were case studies. The age of the participants ranged from 28 to 45 years. The information on participants that was recorded included findings related to blood biochemistry, chronic inflammation, and biomarkers. Most of these studies used the International Association of Diabetes Pregnancy Group’s criteria; only one study, from Thailand, chose its own criteria (22). Owing to different methodologies of analysis, the storage temperature of the stool sample varied (–20°C in one study and –80°C in 19 studies). We focused on the differences emerging when comparing GDM patients with healthy people. One article divided participants into four groups according to their blood pressure and lipid measurement (20). Therefore, we took only the GDM group and the healthy group into consideration. One article did not exclude those who had probiotics or antibiotic treatment during pregnancy (23). Only two articles included the sample size calculation, which makes these studies more reliable than the others (18, 28) (**Supplementary Table 4**).

### 3.2 Quality of included studies

**Figure 2:**
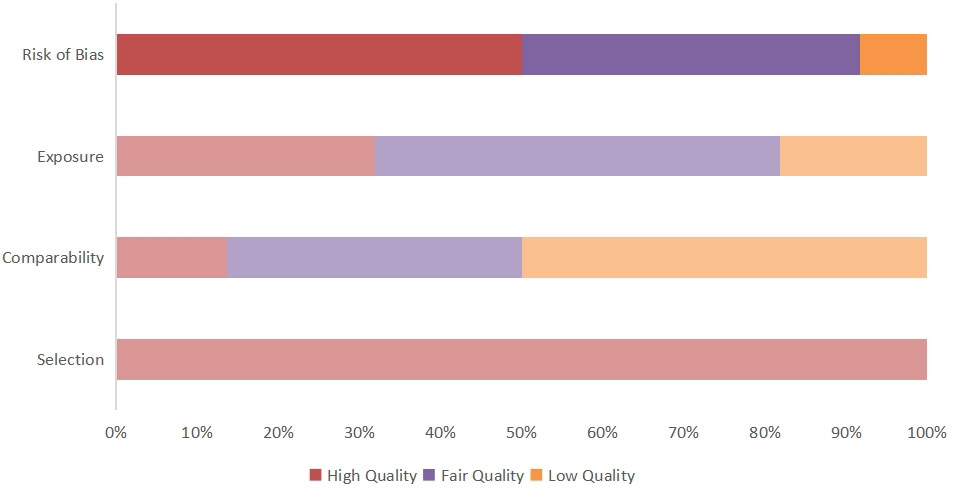
Quality score of included articles calculated using the NOS. The overall article quality score and risk of publication bias are obtained by calculating three indicators.

Quality was assessed using the NOS. Each study was evaluated based on three criteria, namely selection of the participants, comparability of groups (i.e., absence of confounder bias), and exposure (measurement of outcome influencing). Ten articles were assessed as being of fair quality and 12 as being of good quality, no studies were of poor quality The detailed scores for every study are shown in **Figure 2**.

### 3.3 GDM detection and study criteria

In general, GDM is diagnosed at 24–28 gestational weeks, using established criteria from the International Association of Diabetes and Pregnancy Study Groups (IADPSG) based on the results of a standard 2-h, 75-g oral glucose tolerance test (OGTT) (29). Pregnant women are diagnosed with GDM if one or more glucose levels is elevated, as follows: fasting ≥ 5.1 mmol/L, 1 h ≥ 10.0 mmol/L, and 2 h ≥ 8.5 mmol/L.

The basic demographics and clinical characteristics of the participants are shown in **Supplementary Table 3**. The included studies involved 965 participants with GDM and 1,508 healthy people. Only four studies recorded and accounted for pre-study body mass index (BMI) in order to obtain more objective results (18, 19, 28, 30). Participants with other metabolic diseases or who had been treated with antibiotics or probiotics were excluded.

### 3.4 Methodological characteristics of selected articles about stool sample

Stool samples were generally stored at –80°C, except in one study, in which the samples were stored at –20°C (18). Studies used a wide range of DNA extraction kits. Only the QIAamp Fast DNA StoolMini Kit (Portsmouth, NH, USA) was used more than twice. Target DNA sequencing in the V3–V4 region was the most common technique, used in 15 studies, whereas four studies (20, 21, 31, 32) sequenced in the V4 region, one (23) sequenced in the V1–V2 region, and one (26) sequenced in the V6–V8 region. There were four criteria for defining clustering of OTUs, namely 95% OTUs (1/22), 97% OTUs (13/22), 99% OTUs (5/22), and 100% OTUs (1/22). Taxonomy annotation was conducted mostly with an RDP (Ribosomal Database Project) classifier trained on the SILVA (6/20), RDP (3/22), and Greengenes (5/22) databases. Specific information is shown in **Supplementary Table 4**.

### 3.5 Alpha diversity and beta diversity

Diversities are commonly known as alpha diversity and beta diversity. They are used to describe the species composition of the gut microbiota, which may be considered a significant factor influencing outcomes.

**Figure 3:**
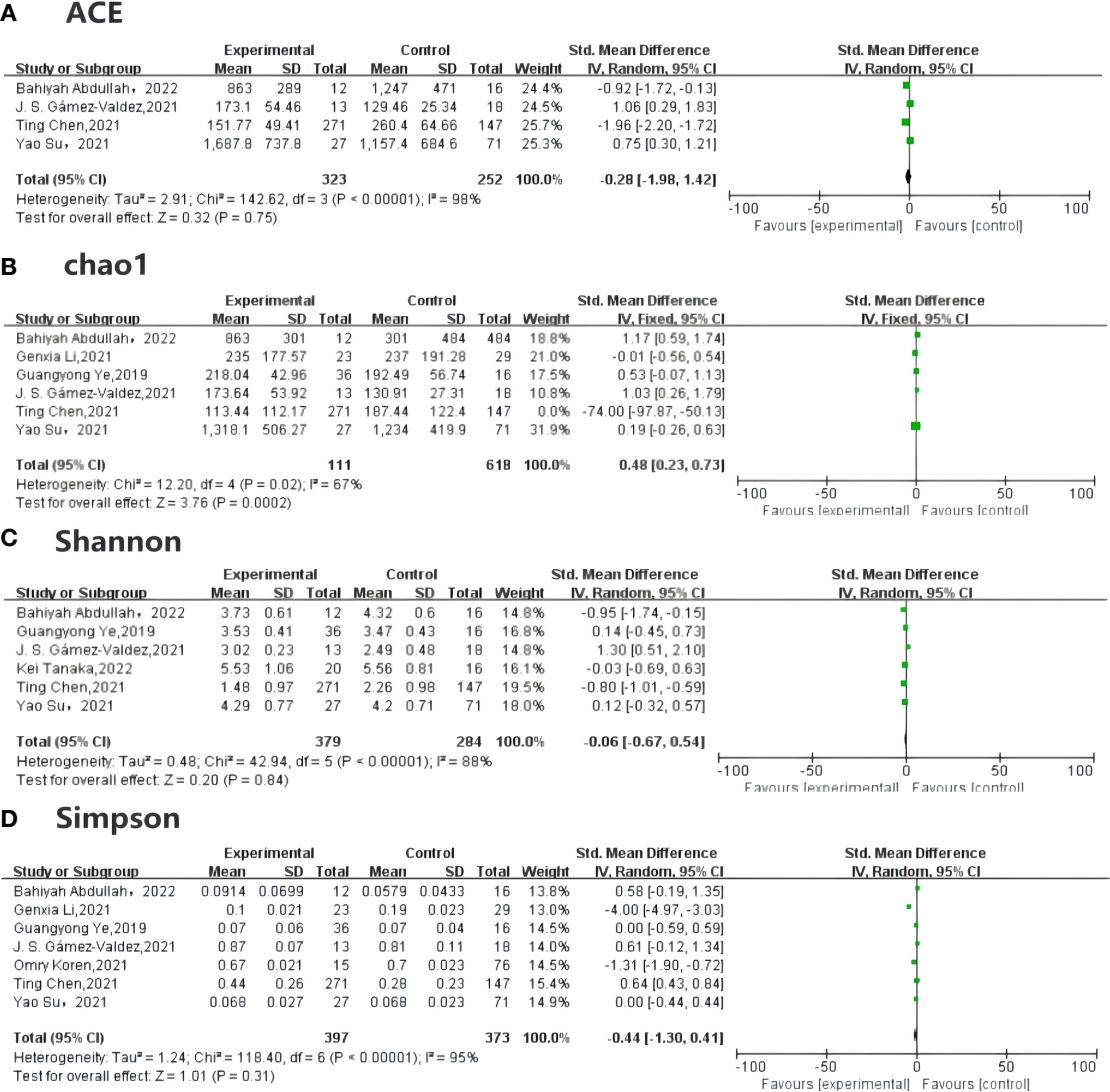
Forest plot of randomized controlled trials comparing the alpha diversity between GDM and NGDM. **(A)** plot means SDs of ACE, **(B)** plot described the Chao1between articles, and **(C)** plot indicates the result for Shannon index, **(D)** plot mentioned the Simpson index, which are all reported in studies. Differences between groups are presented as weights (percentages) and SMD (95% CI). CI, confidence interval; IV, inverse variance; SD, standard deviation; SMD, standardized mean difference.

Alpha diversity can predict both the number of the species and individual distribution, known as richness and evenness, and can be measured by the ACE (abundance-based coverage estimator), Chao1, Simpson, and Shannon indexes. Among the included studies, four (18, 19, 33, 34) compared the ACE in GDM and non-GDM patients [SMD –0.28 (95% CI –1.98 to 1.42), *p* = 0.75, *I*^2^ = 98%]. Six studies (18, 19, 24, 33–35) reported the Chao1 index [SMD 0.48 (95% CI 0.23 to 0.73), *p* < 0.05, *I*^2^ = 67%] for quality assessment. The Shannon index [SMD –0.06 (95% CI –0.67, 0.94), *p* = 0.84, *I*^2^ = 88%] was provided in six studies (18, 19, 28, 33–35) and the Simpson index in seven studies (18, 19, 23, 24, 33–35) [SMD –0.44 (95% CI –1.30 to 0.41), *p* = 0.31, *I*^2^ = 95%]. The indexes were the same in each group (**Figure 3**).

The result for the heterogeneity assessment was not as good as we had anticipated. The *I*^2^-value was >50%, indicating strong heterogeneity. The source of this heterogeneity should be further explored. Therefore, we further performed sensitivity analyses, omitting each study in turn, to ensure accuracy and stability of the results. If, after removing an article from the indexes, both the *I*^2^-value and the *p*-value were stable, indicating that the article has stable sensitivity analysis results.

In addition, to test our hypothesis that the results were reliable, we assessed the risk of publication bias using Egger’s tests, based on the symmetry of a funnel chart. The results indicated no evidence of publication bias, since the *p*-value of each index was above 0.05, showing that the conclusions of the meta-analysis were relatively robust. These results can be seen in **Supplementary Figure 1**.

As for beta diversity,17 articles reported beta diversity, of which 11 used principal component analysis (PCA) and 10 reported results using principal coordinate analysis (PCoA). We were unable to conduct a robust analysis of beta diversity because results were mostly provided in graphical form, rather than as specific data.

### 3.6 Subgroup analysis

Owing to the significant statistical heterogeneity encountered in the analysis, several subgroup analyses were conducted separately. Analyses of classifications of gut microbiome and dietary intakes were carried out provided these were reported in at least two articles.

Subgroup analysis of the gut microbiome at genus level showed that the abundances of *Blautia* [SMD 0.36 (95% CI 0.02 to 0.71), *p* = 0.04, *I*^2^ = 0%] and *Collinsella* [SMD –4.18 (95% CI –8.73 to 0.38), *p* = 0.38, *I*^2^ = 78.9%] were significantly higher in the GDM group than in the control group. There were no differences in-between groups in the abundances of *Clostridium* [SMD –0.47 (95% CI –0.92 to –0.01), *p* = 0.187, *I*^2^ = 42.4%] and *Faecalibacterium* [SMD –0.19 (95% CI –0.42 to 0.04), *p* = 0.971, *I*^2^ = 0%].

Similarly, subgroup analysis showed that high fiber intake, compared with lower fiber intake, protects against GDM [SMD –0.96 (95% CI –0.95 to –0.43), *p* < 0.05, *I*^2^ = 92%], whereas there were no differences between patients and control participants in energy intake [SMD –0.46 (95% CI –1.84 to 0.93), *p* = 0.52, *I*^2^ = 97%], cereal intake [SMD –0.31 (95% CI –0.92 to 0.29), *p* = 0.31, *I*^2^ = 86%], meat intake [SMD –0.08 (95% CI –0.31 to 0.15), *p* = 0.15, *I*^2^ = 93%], or milk intake [SMD –0.66 (95% CI –1.55 to 0.24), *p* = 0.15, I^2^ = 93%], although there remained considerable heterogeneity in all analyses. Specific results and data can be viewed in **Table 1**.

**Table 1:** Subgroup analysis of effect of gut microbiota and dietary intake.

Subgroup	No. of studies	Effect (95% CI)	Coherence		*I^2^*	Egger
			*Q*-value	*p*-value		
*Blautia*	3	0.36 (0.02 to 0.71)	2.72	0.040*	0%	0.332
*Collinsella*	2	–4.18 (–8.73 to 0.38)	2.08	0.038*	78.9%	–
*Clostridium*	2	–0.47 (–0.92 to –0.01)	1.31	0.187	42.4%	–
*Faecalibacterium*	2	–0.19 (–0.42 to 0.04)	0.97	0.332	0%	–
Diet						
Energy intake	4	–0.46 (–1.84 to 0.93)	5.80	0.52	97%	0.448
Cereal intake	4	–0.31(–0.92 to 0.29)	1.60	0.31	86%	0.515
Meat intake	3	–0.08 (–0.31 to 0.15)	0.83	0.48	0.0%	0.824
Milk intake	4	–0.66 (–1.55 to 0.24)	2.79	0.15	93%	0.511
Fiber intake	3	–0.96 (–0.95 to –0.43)	22.03	0.028*	92%	0.990

*Significant difference at a p-value of <0.05.

### 3.7 Differences in taxa abundance in the gut microbiota during pregnancy

The current study compared the gut microbiota in patients with GDM and healthy participants. Nine (18, 19, 21, 23, 24, 27, 30, 34, 36) articles analyzed the taxa at the phylum level. Three (18, 19, 24) articles mentioned *Firmicutes* and concluded that they were less abundant in GDM patients, whereas two (30, 36) articles reported the opposite results. *Bacteroidetes* are more abundant in GDM patients than in participants without DGM. Two articles (23, 34) reported that the abundance of *Actinobacteria* was lower in GDM patients than in those without GDM. Four studies (24, 34, 36, 37) reported that the abundance of *Proteobacteria* was higher in GDM patients than in healthy controls. Only one study (23) reported the opposite. Three studies (24, 27, 30) compared *Firmicutes*/*Bacteroides*, two of which (24, 30) found that their abundance was higher in the GDM group.

At the class level, one (21) study mentioned that *Rothia*, *Actinomyces*, *Bifidobacterium*, *Adlercreutzia*, and *Coriobacteriaceae* from *Actinobacteria* were reduced in the GDM population.

At the family level, there were slight differences between studies. Both *Veillonellaceae* (21, 37) and *Prevotella* group 9 (30, 37) were increased in GDM patients, whereas *Lachnospiraceae* and *Ruminococcaceae* (24) were decreased. Some studies drew controversial conclusions as regard *Streptococcaceae*, *Enterobacteriaceae*, *Lachnospira*, *Clostridiales*, *Clostridia*, and *Firmicutes*. Two (24, 36) studies found that, at the family level, members of the family *Clostridiales* were increased in GDM patients.

Studies reporting findings at the genus level were the most common. We found enrichment of *Ruminococcaceae*, *Lactococcus*, *Escherichia*, *Lachnospiraceae*, *Clostridia*, *Alistpes*, *Firmicutes*, and *Phascolarctobacterium*. Results regarding *Streptococcus* and *Bacteroidetes* varied. We also found enrichment of *Coprococcus*, *Staphylococcus*, *Oscillospira*, Burkholderiales, *Akkermansia*, *Prevotella* group 9, and *Faecalibacterium* in participants without GDM. Abundances of *Blautia*, *Staphylococcus*, *Sutterella*, *Oscillospira*, *Enterococcus*, and *Lactobacillus* also varied, and this needs further study.

We performed further random forest analysis of four dominant bacteria. *Proteobacteria* are relatively abundant in GDM [SMD –0.44 (95% CI –1.3 to 0.41), *p* = 0.31, *I*^2^ = 95%], whereas the abundances of *Actinobacteria* [SMD –1.64 (95% CI –2.32 to –0.95), *p* < 0.05, *I*^2^ = 94%], *Bacteroides* [SMD 7.96 (95% CI –7.77 to 23.69), *p* = 0.32, *I*^2^ = 100%], *Firmicutes* [SMD 0.25 (95% CI –0.01 to 0.51), *p* = 0.06, *I*^2^ = 49%], and *Proteobacteria* [SMD 1.10 (95% CI –1.59 to 3.80), *p* = 0.42, *I*^2^ = 99%] did not differ significantly between the two groups.

### 3.8 Types of gut microbiota and their impacts on GDM

**Figure 4:**
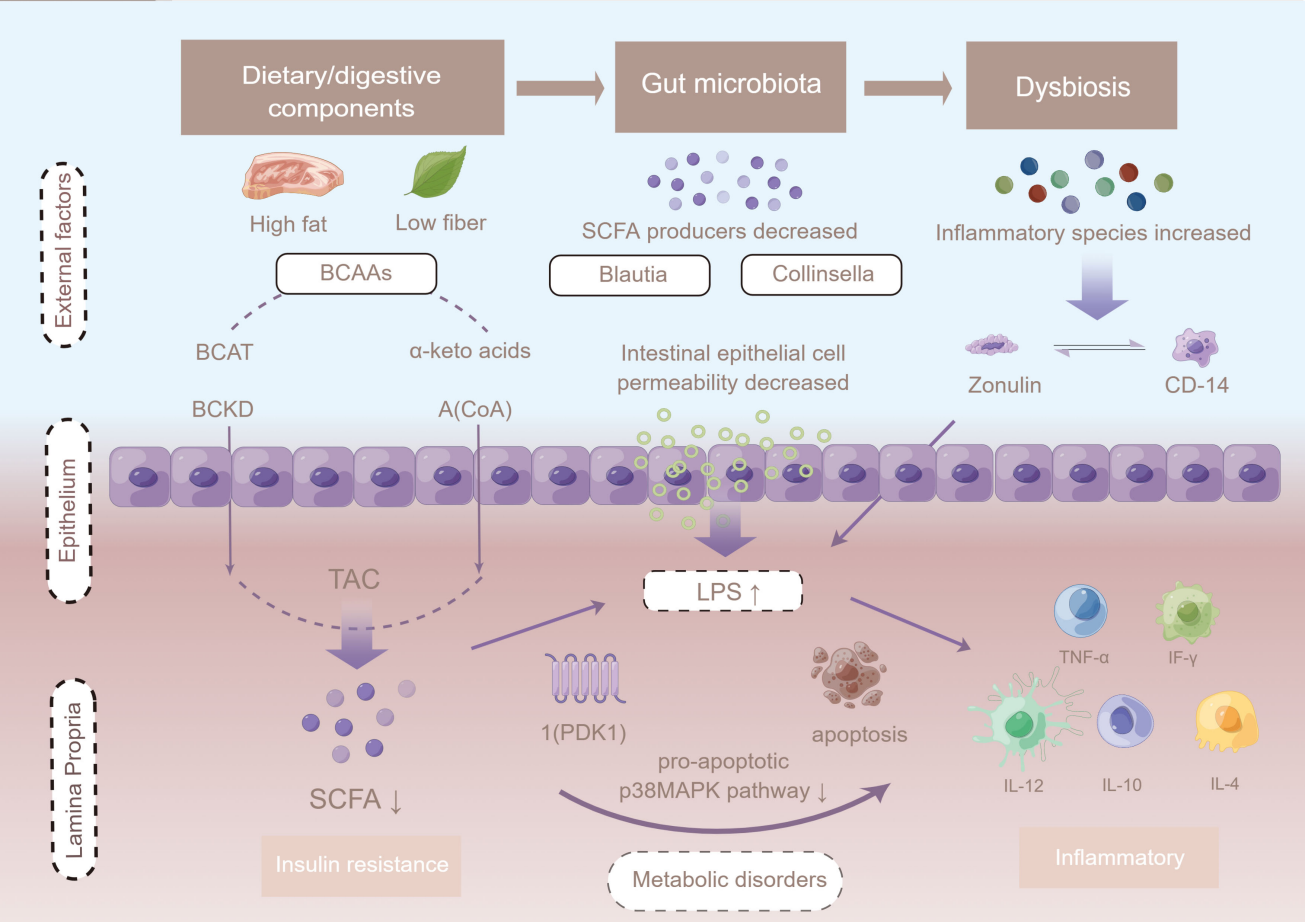
The potential mechanism linking gut dysbiosis and GDM. Intestinal permeability is regulated by dietary factors and the zonulin pathway. Cells in the basal layer of the intestinal epithelium secrete zonulin and bind to initiate complex intracellular signaling pathways, allowing phosphorylation of tight junctions. When LPS is ready to enter the advocate circulation, it increases absorption, and forms a complex with LBP that further binds CD14 released from monocytes, leading to the production of pro-inflammatory cytokines mediated by the MD2/TLR4 receptor complex, such as TNF-α, interleukin 1 (IL-1), and interleukin 6 (IL-6). LPS infiltrates peripheral adipose tissue and binds to TLRs, thus activating the adaptor proteins MyD-88, IRAK, TAK1, and TRAF6, and triggering macrophage infiltration and up-regulation of inflammatory pathways. Up-regulation of JNK/IKKβ/NF-κB may increase serine phosphorylation of IRS-1 + Ser307, resulting in PI3-K inhibition and Akt down-regulation of Ser473. Figure has been obtained for use of copyrighted material from Figuer (https://www.figdraw.com/, registered ID: 533420148).

To further explore the potential correlations of key clinical indexes with altered gut microbiome in GDM, correlation analyses were performed using Spearman analysis. The results showed that phylum *Bacteroidetes* was positively associated with 1hPG, whereas *Proteobacteria*, *Verrucomicrobiota*, and *Actinobacteria* were all negatively associated with 1-hour plasma glucose level (1hPG) levels. Analysis at the genus level revealed a negative association between *Ruminococcaceae* UCG014 and 1hPG, but a positive association between *Ruminococcaceae* UCG014 and high-density lipoprotein (HDL) levels (36). The genus *Akkermansia* was negatively correlated with 1hPG and positive correlated with HDL levels. According to Chen et al., genera of *Clostridiales*, *Ruminococcaceae* and *Lachnospiraceae*, within the phylum *Firmicutes*, were significantly negatively correlated with at least one OGTT value (19). An unassigned genus of *Enterococcaceae* within the phylum *Firmicutes*, the genus *Atopobium* within the phylum *Actinobacteria* and the genus *Sutterella* within the phylum *Proteobacteria* were significantly positively associated with 1-h or 2-h OGTT values (**Supplementary Table 5**; **Figure 4**).

Indexes of inflammation, fecal calprotectin (FCALP), lipopolysaccharide (LPS), lipopolysaccharide-binding protein (LBP), and fecal LPS (FLPS), were reported in two (20, 26) studies. Levels of zonulin, FCALP, LPS, LBP, and FLPS were higher in GDM patients than in those without GDM, and the results were statistically significant. Cui et al. reported that *Enterococcus* and *Vagococcus* are in direct proportion to FCALP and LPS, *Streptococcus* is inversely proportional to LBP, and *Staphylococcus* is directly proportional to FLPS (20). Women’s diet, including total energy and fiber intake, remained unchanged between sampling times.

Four articles reported Kyoto Encyclopedia of Genes and Genomes (KEGG) pathway analysis. The conclusions drawn in each article vary widely. Chen et al. identified several microbial gene pathways including the glycan biosynthesis and vitamin metabolic pathways (19). Li et al. showed that the species of gut microbes found in normoglycemic pregnant women (NOR) and GDM are involved in cell wall/membrane/envelope biogenesis, organic ion transport and metabolism, post-translational modifications, protein turnover, chaperones, transcription, unknown function, intracellular transport, secretion, and vesicular transport (24). Su et al. found a positive relationship between *Bacteroides* species enriched in patients with GDM, and amino sugar and nucleotide sugar metabolism (34). Wang et al. found that the predicted metagenome of women who developed GDM was enriched in organisms involved in starch and sucrose metabolism, whereas those implicated in lysine biosynthesis and nitrogen metabolism were reduced (37).

## 4 Discussion

To the best of our knowledge, this is the first article to systematically analyze how enterobacterial differences affect metabolic health in GDM patients and healthy individuals. Consistent evidence has shown that the composition of the gut microbiota is specifically altered in GDM. We found differences between groups in the abundance of microorganisms. These suggested that an increase in *Blautia* and a reduction in *Clostridium* may make a huge contribution, and thus may provide bacterial targets to prevent or treat GDM by reconstructing the homeostasis of the gut microbiota. Future studies in this area are warranted. The primary purpose of this study was to determine the validations between the participant groups, which may provide insights into possible mechanistic links between the gut microbiota and GDM and the pathway leading to disease development. The available results suggested an association between microbial composition and disease. Despite differences in lateral suction patterns, diversity and composition results were generally consistent between studies. Alpha diversity is widely used for measuring the richness and evenness of the gut microbiome. Our meta-analysis demonstrated a significant association between the presence of GDM and reductions in diversity indexes, suggesting that species richness was reduced in the affected individuals. Although alpha diversity has been shown to be a marker of chronic diseases, such as T2DM, colorectal cancer, and non-alcoholic fatty liver disease (NAFLD), multiple studies of the gut microbiome in patients withT2DM have shown no statistically difference in alpha diversity among well-matched participants (38–42).

Some studies found that alpha diversity was slightly lower, but not significantly reduced, in T2DM patients than in healthy participants (41). Occasionally, richness (as measured by the Shannon and Simpson indexes), which has been associated with elevated insulin resistance, shows an overlapping trend in GDM patients and healthy individuals. However, our meta-analysis results showed that there were no significant differences in diversity or richness between GDM patients and healthy individuals, indicating that alpha diversity may not be a hallmark indicator distinguishing between individuals with and without GDM. In contrast to alpha diversity, beta diversity differed in the GDM group and the control group participants, highlighting the fact that the profile of gut microbiota was altered in GDM. Further studies at the phylum level identified the taxa of the gut microbiota whose abundance was altered by GDM and suggested that an increase in *Bacteroides* and *Proteobacteria* and a reduction in *Actinobacteria* may contribute to GDM.

The phyla identified after *in vitro* fecal fermentation were mainly *Firmicutes*, *Bacteroides*, *Actinobacteria*, and *Proteobacteria*. In addition, this review found that the *Firmicutes*/*Bacteroidetes* ratio was significantly higher in GDM patients than in healthy control participants. *Firmicutes* and *Bacteroides* are the two dominant bacterial groups in the gut. They can maintain energy balance in the host by participating in the metabolism of fats and bile acids. The *Firmicutes*/*Bacteroides* ratio is commonly used as a marker of low-grade systemic inflammation in obesity and insulin resistance and as an indicator of gut microbiota composition in different individuals (43–46). Similar to other findings (47, 48),the *Firmicutes*/*Bacteroides* ratio was higher in GDM patients, indicating that it is a sensitive indicator enabling patients with GDM to be distinguished from those who do not have GDM. Animal experiments have shown that colonization of the normal gastrointestinal tract, as shown in the cultivate experiments of *Bacteroides* species and meditated through toll-like receptors (TLRs) and other specific host–microbe interactions is a result of recognition and selection by the host immune system (49). In general, insulin resistance is associated with a higher *Firmicutes*/*Bacteroides* ratio and a reduction in the number of butyrate-producing bacteria, while *Bacteroidetes*, as the most stable component of the gastrointestinal microbiota in healthy adults, contains most genera that produce butyrate (50). Butyrate is considered a health-promoting molecule because it can increase insulin sensitivity, exert anti-inflammatory activity, regulate energy metabolism, and increase leptin gene expression (51–54). Propionate in the colon stimulates the release of glucagon-like peptide 1 (GLP-1) and peptide YY (PYY) from L-enteroendocrine cells, thereby suppressing appetite (55). It may also reach the portal circulation and get captured by the liver tissue, where it participates in hepatic gluconeogenesis and reduces the expression of enzymes involved in fatty acid and cholesterol synthesis (56).

*Blautia* has shown a significant negative correlation with many diseases, including type 1 diabetes mellitus (T1DM), obesity, and Crohn’s disease (57, 58). As a risk marker of adiposity and of cardiovascular and metabolic disease, *Blautia* abundance has been shown to be inversely associated with visceral fat tissue (58). This may be due to a potential anti-inflammatory effect that can reduce the ratio of TNF-α to IL-4 in T2DM patients, balance immunity with anti-inflammation, and help maintain glucose homeostasis, so as to regulate the transduction of insulin signaling, thereby regulating the conduction of insulin signals such as fasting blood glucose (FBG) level (59–61). The abundance of *Blautia* in patients with T1DM is consistent with HbA_1c_ and FBG results (57). This study did in fact find that *Blautia* abundance was slightly increased in GDM patients, which may be because disease was most often diagnosed in the first trimester (62). From the first to the third trimesters, *Blautia* levels gradually declined, resulting in a decrease in butyrate production, stimulating neutrophils and macrophages to release inflammatory factors (63). An increase in inflammatory factors is associated with low levels of fiber intake, which may lead to the metabolism, and thereby cause dysbiosis and aggravated inflammation (64).

The genus *Collinsella* from the family *Lachnospiraceae* is often described as a strictly anaerobic pathobiont that produces lactate, which is often correlated with SCFAs (65). The decreased abundance of this taxon is associated with the health status of patients suffering from T1DM and T2DM (41). This genus of bacteria seems to be stimulated by a low-fiber diet, which could be observed in GDM patients. Both *in vitro* and animal experiments have concluded that SCFAs modulate intestinal inflammation by improving transepithelial resistance, altering various signaling pathways, and inhibiting pro-inflammatory cytokines, while up-regulating anti-inflammatory cytokines (66, 67). SCFAs stimulate glucagon production and signal the hypothalamus as a mechanism of diabetes. Butyrate suppresses the production of pro-inflammatory cytokines, for example TNF-α, IL-12, and interferon γ (IF-γ), and up-regulates the production of anti-inflammatory IL-10 by monocytes, thereby producing anti-inflammatory effects (53). Butyrate has been shown to attenuate LPS-stimulated pro-inflammatory effects (68, 69). Intake of a high-fat and low-fiber diet may alter the normal composition of gut microbiota and dietary fermentation. Alterations in dietary fermentation may lead to excessive production of SCFAs and absorption of energy from the diet. The relative abundance of *Firmicutes* was elevated, while the abundances of *Actinobacteria* and *Bacteroidetes* were lower, in women with GDM (70). This “gut microbiota signature” is similar to the phenotype of metabolic disorder, which is mainly due to the obese phenotype (71). Moreover, metabolic pathways mentioned in research are those linked with carbohydrate metabolism, such as glycolysis/gluconeogenesis, starch and sucrose metabolism, and galactose metabolism, which can be enriched in women with GDM (64). The results of our investigation of dietary status showed no difference in energy intake between the two groups of individuals (i.e., those with and without GDM), but that dietary fiber intake was lower in the GDM group in the non-GDM population, suggesting a possible mechanism of GDM. Butyrate-producing bacteria, such as *Faecalibacterium* and *Akkermansia* responses the same trend as on fiber intake in the context of the entire diet (72, 73). Thus, it is clear that dietary fiber components are enhanced with the microbiota.

Intestinal microbes also regulate the process of absorption of metabolites and endotoxins by affecting intestinal permeability. It has been shown that the development of GDM is associated with an increase in LPS in the intestine during late pregnancy as well as intestinal mucosal injury characterized by elevated levels of serum LPS and streptoglobulin. In this study, the level of inflammatory factors was significantly higher in GDM patients than in those without GDM, which is consistent with specific physiological changes. Intestinal permeability is regulated by dietary factors and the zonulin pathway. Cells in the basal layer of the intestinal epithelium secrete zonulin and bind to initiate complex intracellular signaling pathways, allowing phosphorylation of tight junctions, which in turn leads to an increased permeability (74). When LPS is ready to enter the advocate circulation, it would increase absorption, and form a complex with LBP that further binds CD14 from monocytes (75). This may lead to the production of pro-inflammatory cytokines mediated by the MD2/TLR4 receptor complex, such as TNF-α, IL-1, and IL-6 and LPS, which infiltrate peripheral adipose tissue and bind to TLRs, activating the adaptor proteins MyD-88, IRAK, TAK1, and TRAF6, and as a result triggering macrophage infiltration and up-regulation of inflammatory pathways. Up-regulation of JNK/IKKβ/NF-κB may increase serine phosphorylation of IRS-1 + Ser307, resulting in PI3-K inhibition and Akt down-regulation of Ser473. Reducing acetate Ser473 phosphorylation may impair insulin signaling and reduce glucose uptake in peripheral tissues, leading to hyperglycemia in women with GDM (74, 76).

Previous studies have shown that the efficiency of energy extraction from the diet is correlated with the enrichment of specific metabolic pathways, particularly those involved in carbohydrate transport and utilization (77). Amino acids are also insensitive to insulin action. Isoenzyme branched-chain amino acid aminotransferase (mitochondrial BCAT and cytosolic BCAT) catalyze the first reversible transamination/deamination of branched-chain amino acids (BCAAs) to their corresponding α-ketoacids. They are then combined in order to convert α-ketoglutarate into glutamate; leucine, isoleucine, and valine yield α-ketoisocaproate (KIC), α-keto-β-methylglutarate (KIM), and α-ketoisovalerate (KIV), respectively (78). The second step of BCAA catabolism is mainly regulated by the catalytic activity of the branched-chain ketoacid dehydrogenase (BCKD) complex (79). Branched-chain acyl-coenzyme A (CoA) species are produced from their cognate α-ketoacids (80). Complete metabolism of BCCA species generates cataplerotic metabolites that are subsequently used in the tricarboxylic acid (TCA) cycle for the generation of fuel (i.e., ATP), using lipogenic, ketogenic, or glucogenic substrates (81–83). In addition, down-regulated BCAA catabolic genes prominent in the adipose tissue of participants with elevated BCAA serum concentrations were correlated with high HOMA-IR values (*p* < 0.05) (84). It has been shown that bacteria of the genus *Lactobacillus* help maintain metabolic homeostasis by improving amino acid metabolic pathways in metabolically impaired mice to better compensate for impaired aryl hydrocarbon receptor (AhR) signaling by increasing the availability of intestinal metabolites capable of signaling through the AhR (85). It has been reported from experimental studies that the gut microbiota can directly utilize tryptophan and produce bacteria-derived indole (86, 87). Examples include indolyl sulfate and *p*-cresol sulfate, which stimulate GLP-1 and increase secretion insulin from pancreatic beta cells (83, 88, 89). Similarly, results from the KEGG enrichment analysis showed that by far the most-downregulated pathways were BCAA degradation pathways (74).

This review has several deficiencies. First, the small sample size means that the accuracy of the study is low. Second, most articles did not provide sufficient specific indicators of gut microbial diversity and composition, making accurate quantitative analysis impossible. As a result, the evidence provided by these studies is insufficient. Third, as a result of limited data, we were unable to stratify patients by sampling time, diet, obesity, Asian-European factors, etc. Mechanistic insight into the gut microbiota is still limited, so further study is needed to identify specific biomarkers and the mechanisms by which they cause disease.

## Data availability statement

The original contributions presented in the study are included in the article/**Supplementary Material**. Further inquiries can be directed to the corresponding authors.

## Author contributions

MY, XG and DH wrote the manuscript and researched the data. MY and RH researched data, contributed to discussion, and reviewed the manuscript. SC, ZL, RH, and DZ contributed to the discussion and reviewed the manuscript. All authors read and approved the final version of the manuscript, and take responsibility for the integrity of the data and the accuracy of the data analysis.
