# Supplementary material for: Mechanismbased role of the intestinal microbiota in gestational diabetes mellitus: A systematic review and meta-analysis
Source: Front Immunol. 2023 Mar 3;13:1097853. doi: 10.3389/fimmu.2022.1097853 (PMC10020587; doi:10.3389/fimmu.2022.1097853)
Supplement: Supplementary file 1 [file DataSheet_1.docx]

Supplementary Material

**Supplementary Table 1.** Systematic search detail.

**Supplementary Table 2.** Quality assessment of the included studies using the Newcastle-Ottawa Scale.

**Supplementary Table 3.** Study characteristics of each study focusing on basic data.

**Supplementary Table 4.** Study characteristics of each study focusing on stool sample storage, extraction and analysis.

**Supplementary Table 5.** The relationships between intestinal microbiota and biochemical indicators.

**Supplementary Figure 1.** Funnel plots assessing publication bias.

**Supplementary Table 1. Systematic search detail.**

- 1. **Embase search stratergy for 34**

| **Sequence** | **Search words** | **Hits** |
| --- | --- | --- |
| #1 | pregnancy diabetes mellitus':ab,ti OR 'pregnancy in diabetic':ab,ti OR 'pregnancy in diabetes':ab,ti OR 'pregnancy in diabete':ab,ti OR 'pregnancy':ab,ti OR 'pregnent':ab,ti | 576761 |
| #2 | alkaline diet':ab,ti OR 'artificial diet':ab,ti OR 'atkins diet':ab,ti OR 'carbohydrate diet':ab,ti OR 'carbohydrate loading diet':ab,ti OR 'cariogenic diet':ab,ti OR 'casein free diet':ab,ti OR 'cereal-based diet':ab,ti OR 'dash diet':ab,ti OR 'elemental diet':ab,ti OR 'elimination diet':ab,ti OR 'experimental diet':ab,ti OR 'fad diet':ab,ti OR 'fiber free diet':ab,ti OR 'gluten free diet':ab,ti OR 'healthy diet':ab,ti OR 'high calorie diet':ab,ti OR 'high fiber diet':ab,ti OR 'high glycemic index diet':ab,ti OR 'high salt diet':ab,ti OR 'intuitive eating':ab,ti OR 'ketogenic diet':ab,ti OR 'lipid diet':ab,ti OR 'lactose free diet':ab,ti OR 'liquid diet':ab,ti OR 'low calorie diet':ab,ti OR 'low carbohydrate diet':ab,ti OR 'low fiber diet':ab,ti OR 'low fodmap diet':ab,ti OR 'low glycemic index diet':ab,ti OR 'low iodine diet':ab,ti OR 'low residue diet':ab,ti OR 'macrobiotic diet':ab,ti OR 'mediterranean diet':ab,ti OR 'nordic diet':ab,ti OR 'obesogenic diet':ab,ti OR 'okinawan diet':ab,ti OR 'paleolithic diet':ab,ti OR 'protein diet':ab,ti OR 'raw food diet':ab,ti OR 'soft diet':ab,ti OR 'unhealthy diet':ab,ti OR 'vegetarian diet':ab,ti OR 'western diet':ab,ti OR 'diet':ab,ti OR 'dietary':ab,ti | 698799 |
| #3 | nutrition':ab,ti OR 'adolescent nutrition':ab,ti OR 'carbon source':ab,ti OR 'child nutrition':ab,ti OR 'dietary intake':ab,ti OR 'dietary pattern':ab,ti OR 'fat content':ab,ti OR 'fat load':ab,ti OR 'feeding behavior':ab,ti OR 'food access':ab,ti OR 'food availability':ab,ti OR 'food chain':ab,ti OR 'food frequency questionnaire':ab,ti OR 'food intake':ab,ti OR 'geriatric nutrition':ab,ti OR 'glycemic index':ab,ti OR 'glycemic load':ab,ti OR 'immunonutrition':ab,ti OR 'maternal nutrition':ab,ti OR 'nutrient availability':ab,ti OR 'nutrient concentration':ab,ti OR 'nutrient content':ab,ti OR 'nutrient cycle':ab,ti OR 'nutrient cycling':ab,ti OR 'nutrient dynamics':ab,ti OR 'nutrient limitation':ab,ti OR 'nutrient loading':ab,ti OR 'nutrient management':ab,ti OR 'nutrient supply':ab,ti OR 'nutrient uptake':ab,ti OR 'nutrition education':ab,ti OR 'nutrition labeling':ab,ti OR 'nutritional assessment':ab,ti OR 'nutritional counseling':ab,ti OR 'nutritional health':ab,ti OR 'nutritional requirement':ab,ti OR 'nutritional status':ab,ti OR 'nutritional tolerance':ab,ti OR 'nutritional value':ab,ti OR 'personalized nutrition':ab,ti OR 'plant nutrition':ab,ti OR 'sport nutrition':ab,ti OR 'water deprivation':ab,ti | 475305 |
| #4 | life style':ab,ti OR 'life styles':ab,ti OR 'lifestyle':ab,ti OR 'lifestyles':ab,ti OR 'life style?induced illness':ab,ti OR 'lifestyle factors':ab,ti OR 'factor, lifestyle':ab,ti OR 'lifestyle factor':ab,ti | 183389 |
| #5 | physical fitness':ab,ti OR 'fitness, physical':ab,ti | 14380 |
| #6 | exercise':ab,ti OR 'exercises':ab,ti OR 'physical activity':ab,ti OR 'activities, physical':ab,ti OR 'activity, physical':ab,ti OR 'physical activities':ab,ti OR 'exercise, physical':ab,ti OR 'exercises, physical':ab,ti OR 'physical?exercise':ab,ti OR 'physical exercises':ab,ti OR 'acute?exercise':ab,ti OR 'acute exercises':ab,ti OR 'exercise, acute':ab,ti OR 'exercises, acute':ab,ti OR 'exercise, isometric':ab,ti OR 'exercises, isometric':ab,ti OR 'isometric exercises':ab,ti OR 'isometric?exercise':ab,ti OR 'exercise, aerobic':ab,ti OR 'aerobic?exercise':ab,ti OR 'aerobic exercises':ab,ti OR 'exercises, aerobic':ab,ti OR 'exercise?training':ab,ti OR 'exercise?trainings':ab,ti OR 'training,?exercise':ab,ti OR 'trainings,?exercise':ab,ti | 575752 |
| #7 | motor activity':ab,ti OR 'activities, motor':ab,ti OR 'activity, motor':ab,ti OR 'motor activities':ab,ti | 21584 |
| #8 | probiotic agent':ab,ti OR 'probiotic':ab,ti OR 'probiotics':ab,ti | 39786 |
| #9 | cohort studies':ab,ti OR 'case reports':ab,ti OR 'cohort study':ab,ti OR 'studies, cohort':ab,ti OR 'study, cohort':ab,ti OR 'concurrent studies':ab,ti OR 'studies, concurrent':ab,ti OR 'concurrent study':ab,ti OR 'study, concurrent':ab,ti OR 'closed?cohort studies':ab,ti OR 'cohort studies, closed':ab,ti OR 'closed cohort study':ab,ti OR 'cohort study, closed':ab,ti OR 'study, closed cohort':ab,ti OR 'studies, closed cohort':ab,ti OR 'birth?cohort studies':ab,ti OR 'birth cohort study':ab,ti OR 'cohort studies, birth':ab,ti OR 'cohort study, birth':ab,ti OR 'studies, birth cohort':ab,ti OR 'study, birth cohort':ab,ti OR 'analysis, cohort':ab,ti OR 'analyses, cohort':ab,ti OR 'cohort analyses':ab,ti OR 'cohort analysis':ab,ti OR 'cohort studies, historical':ab,ti OR 'historical?cohort studies':ab,ti OR 'cohort study, historical':ab,ti OR 'historical cohort study':ab,ti OR 'study, historical cohort':ab,ti OR 'studies, historical cohort':ab,ti OR 'incidence studies':ab,ti OR 'incidence study':ab,ti OR 'studies, incidence':ab,ti OR 'study, incidence':ab,ti OR 'prospective cohort':ab,ti OR 'case controls':ab,ti OR 'case-controls':ab,ti OR '(case control':ab,ti | 733817 |
| #10 | 16srna':ab,ti OR 'metagenomics':ab,ti OR 'community genomics':ab,ti OR 'genomics, community':ab,ti OR 'population genomics':ab,ti OR 'genomics, population':ab,ti OR 'environmental genomics':ab,ti OR 'gastrointestinal microbiome':ab,ti OR 'gastrointestinal microbiomes':ab,ti OR 'microbiome, gastrointestinal':ab,ti OR 'gut microbiomes':ab,ti OR 'gut microbiome':ab,ti OR 'microbiome, gut':ab,ti OR 'gut microflora':ab,ti OR 'microflora, gut':ab,ti OR 'gut microbiota':ab,ti OR 'gut microbiotas':ab,ti OR 'gastrointestinal flora':ab,ti OR 'microbiota, gut':ab,ti OR 'flora, gastrointestinal':ab,ti OR 'gut flora':ab,ti OR 'flora, gut':ab,ti OR 'gastrointestinal microbiota':ab,ti OR 'gastrointestinal microbiotas':ab,ti OR 'microbiota, gastrointestinal':ab,ti OR 'gastrointestinal microbial community':ab,ti OR 'gastrointestinal microbial communities':ab,ti OR 'microbial community, gastrointestinal':ab,ti OR 'gastrointestinal microflora':ab,ti OR 'microflora, gastrointestinal':ab,ti OR 'gastric microbiome':ab,ti OR 'gastric microbiomes':ab,ti OR 'microbiome, gastric':ab,ti OR 'intestinal microbiomes':ab,ti OR 'intestinal microbiome':ab,ti OR 'microbiome, intestinal':ab,ti OR 'intestinal microbiota':ab,ti OR 'intestinal microbiotas':ab,ti OR 'microbiota, intestinal':ab,ti OR 'intestinal microflora':ab,ti OR 'microflora, intestinal':ab,ti OR 'intestinal flora':ab,ti OR 'flora, intestinal':ab,ti OR 'enteric bacteria':ab,ti OR 'bacteria, enteric':ab,ti | 81874 |
| #11 | #2 OR #3 OR #4 OR #5 OR #6 OR #7 OR #8 | 1688011 |
| #12 | #1 AND #9 AND #10 AND #11 AND [english]/lim AND [07-07-2012]/sd NOT [08-07-2022]/sd | 34 |

- 1. **Pubmed search stratergy for 155**

| **Sequence** | **Search words** | **Hits** |
| --- | --- | --- |
| #1 | ("Probiotics"[Mesh]) OR (("Probiotics"[Mesh]) OR (Probiotic[Title/Abstract])) | 31259 |
| #2 | (((((("Cohort Studies"[Mesh]) OR (((((((((((((((((((((((((((((((((Cohort studies[Title/Abstract]) OR (Studies, Cohort[Title/Abstract])) OR (Study, Cohort[Title/Abstract])) OR (Concurrent Studies[Title/Abstract])) OR (Studies, Concurrent[Title/Abstract])) OR (Concurrent Study[Title/Abstract])) OR (Study, Concurrent[Title/Abstract])) OR (Closed Cohort Studies[Title/Abstract])) OR (Cohort Studies, Closed[Title/Abstract])) OR (Closed Cohort Study[Title/Abstract])) OR (Cohort Study, Closed[Title/Abstract])) OR (Study, Closed Cohort[Title/Abstract])) OR (Studies, Closed Cohort[Title/Abstract])) OR (Birth Cohort Studies[Title/Abstract])) OR (Birth Cohort Study[Title/Abstract])) OR (Cohort Studies, Birth[Title/Abstract])) OR (Cohort Study, Birth[Title/Abstract])) OR (Studies, Birth Cohort[Title/Abstract])) OR (Study, Birth Cohort[Title/Abstract])) OR (Analysis, Cohort[Title/Abstract])) OR (Analyses, Cohort[Title/Abstract])) OR (Cohort Analyses[Title/Abstract])) OR (Cohort Analysis[Title/Abstract])) OR (Historical Cohort Studies[Title/Abstract])) OR (Cohort Studies, Historical[Title/Abstract])) OR (Cohort Study, Historical[Title/Abstract])) OR (Historical Cohort Study[Title/Abstract])) OR (Study, Historical Cohort[Title/Abstract])) OR (Studies, Historical Cohort[Title/Abstract])) OR (Incidence Studies[Title/Abstract])) OR (Incidence Study[Title/Abstract])) OR (Studies, Incidence[Title/Abstract])) OR (Study, Incidence[Title/Abstract]))) OR ("Case Reports" [Publication Type]) OR (((Case Study[Title/Abstract]) OR (Case Studies[Title/Abstract])) OR (Case Histories[Title/Abstract]))) OR (Prospective cohort[Title/Abstract])) OR (case controls[Title/Abstract])) OR (case-controls[Title/Abstract])) OR (case control[Title/Abstract]) | 4791268 |
| #3 | (("Diet"[Mesh]) OR (Diets[Title/Abstract])) OR (dietary[Title/Abstract]) OR nutrition[Title/Abstract] OR ((((((("Life Style"[Mesh]) OR (Life Styles[Title/Abstract])) OR (Lifestyle[Title/Abstract])) OR (Lifestyles[Title/Abstract])) OR (Life Style Induced Illness[Title/Abstract])) OR (Lifestyle Factors[Title/Abstract])) OR (Factor, Lifestyle[Title/Abstract])) OR (Lifestyle Factor[Title/Abstract]) OR ("Physical Fitness"[Mesh]) OR (Fitness, Physical[Title/Abstract]) OR ((((((((((((((((((((((((("Exercise"[Mesh]) OR (Exercises[Title/Abstract])) OR (Physical Activity[Title/Abstract])) OR (Activities, Physical[Title/Abstract])) OR (Activity, Physical[Title/Abstract])) OR (Physical Activities[Title/Abstract])) OR (Exercise, Physical[Title/Abstract])) OR (Exercises, Physical[Title/Abstract])) OR (Physical Exercise[Title/Abstract])) OR (Physical Exercises[Title/Abstract])) OR (Acute Exercise[Title/Abstract])) OR (Acute Exercises[Title/Abstract])) OR (Exercise, Acute[Title/Abstract])) OR (Exercises, Acute[Title/Abstract])) OR (Exercise, Isometric[Title/Abstract])) OR (Exercises, Isometric[Title/Abstract])) OR (Isometric Exercises[Title/Abstract])) OR (Isometric Exercise[Title/Abstract])) OR (Exercise, Aerobic[Title/Abstract])) OR (Aerobic Exercise[Title/Abstract])) OR (Aerobic Exercises[Title/Abstract])) OR (Exercises, Aerobic[Title/Abstract])) OR (Exercise Training[Title/Abstract])) OR (Exercise Trainings[Title/Abstract])) OR (Training, Exercise[Title/Abstract])) OR (Trainings, Exercise[Title/Abstract]) OR (((Activities, Motor[Title/Abstract]) OR (Activity, Motor[Title/Abstract])) OR (Motor Activities[Title/Abstract])) OR ("Motor Activity"[Mesh]) | 1255071 |
| #4 | (("Gastrointestinal Microbiome"[Mesh]) OR ((((((((((((((((((((((((((((((((((((Microbiome, Gastrointestinal[Title/Abstract]) OR (Gut Microbiome[Title/Abstract])) OR (Gut Microbiomes[Title/Abstract])) OR (Microbiome, Gut[Title/Abstract])) OR (Gut Microflora[Title/Abstract])) OR (Microflora, Gut[Title/Abstract])) OR (Gut Microbiota[Title/Abstract])) OR (Gut Microbiotas[Title/Abstract])) OR (Microbiota, Gut[Title/Abstract])) OR (Gastrointestinal Flora[Title/Abstract])) OR (Flora, Gastrointestinal[Title/Abstract])) OR (Gut Flora[Title/Abstract])) OR (Flora, Gut[Title/Abstract])) OR (Gastrointestinal Microbiota[Title/Abstract])) OR (Gastrointestinal Microbiotas[Title/Abstract])) OR (Microbiota, Gastrointestinal[Title/Abstract])) OR (Gastrointestinal Microbial Community[Title/Abstract])) OR (Gastrointestinal Microbial Communities[Title/Abstract])) OR (Microbial Community, Gastrointestinal[Title/Abstract])) OR (Gastrointestinal Microflora[Title/Abstract])) OR (Microflora, Gastrointestinal[Title/Abstract])) OR (Gastric Microbiome[Title/Abstract])) OR (Gastric Microbiomes[Title/Abstract])) OR (Microbiome, Gastric[Title/Abstract])) OR (Intestinal Microbiome[Title/Abstract])) OR (Intestinal Microbiomes[Title/Abstract])) OR (Microbiome, Intestinal[Title/Abstract])) OR (Intestinal Microbiota[Title/Abstract])) OR (Intestinal Microbiotas[Title/Abstract])) OR (Microbiota, Intestinal[Title/Abstract])) OR (Intestinal Microflora[Title/Abstract])) OR (Microflora, Intestinal[Title/Abstract])) OR (Intestinal Flora[Title/Abstract])) OR (Flora, Intestinal[Title/Abstract])) OR (Enteric Bacteria[Title/Abstract])) OR (Bacteria, Enteric[Title/Abstract]))) | 71740 |
| #5 | (((((16sRNA[Title/Abstract]) OR ("metagenomics"[Mesh])) OR (Community Genomics[Title/Abstract])) OR (Genomics, Community[Title/Abstract])) OR (Population Genomics[Title/Abstract])) OR (Genomics, Population[Title/Abstract]) | 10721 |
| #6 | ((("Diabetes, Gestational"[Mesh]) OR ((((((Diabetes, Pregnancy-Induced[Title/Abstract]) OR (Diabetes, Pregnancy Induced[Title/Abstract])) OR (Pregnancy-Induced Diabetes[Title/Abstract])) OR (Gestational Diabetes[Title/Abstract])) OR (Diabetes Mellitus, Gestational[Title/Abstract])) OR (Gestational Diabetes Mellitus[Title/Abstract]))) OR (pregnent[Title/Abstract])) OR (pregnency[Title/Abstract]) | 22985 |
| #7 | (((((((("Diabetes, Gestational"[Mesh]) OR ((((((Diabetes, Pregnancy-Induced[Title/Abstract]) OR (Diabetes, Pregnancy Induced[Title/Abstract])) OR (Pregnancy-Induced Diabetes[Title/Abstract])) OR (Gestational Diabetes[Title/Abstract])) OR (Diabetes Mellitus, Gestational[Title/Abstract])) OR (Gestational Diabetes Mellitus[Title/Abstract]))) OR (pregnent[Title/Abstract])) OR (pregnency[Title/Abstract])) AND ((((((16sRNA[Title/Abstract]) OR ("metagenomics"[Mesh])) OR (Community Genomics[Title/Abstract])) OR (Genomics, Community[Title/Abstract])) OR (Population Genomics[Title/Abstract])) OR (Genomics, Population[Title/Abstract]))) AND ((("Gastrointestinal Microbiome"[Mesh]) OR ((((((((((((((((((((((((((((((((((((Microbiome, Gastrointestinal[Title/Abstract]) OR (Gut Microbiome[Title/Abstract])) OR (Gut Microbiomes[Title/Abstract])) OR (Microbiome, Gut[Title/Abstract])) OR (Gut Microflora[Title/Abstract])) OR (Microflora, Gut[Title/Abstract])) OR (Gut Microbiota[Title/Abstract])) OR (Gut Microbiotas[Title/Abstract])) OR (Microbiota, Gut[Title/Abstract])) OR (Gastrointestinal Flora[Title/Abstract])) OR (Flora, Gastrointestinal[Title/Abstract])) OR (Gut Flora[Title/Abstract])) OR (Flora, Gut[Title/Abstract])) OR (Gastrointestinal Microbiota[Title/Abstract])) OR (Gastrointestinal Microbiotas[Title/Abstract])) OR (Microbiota, Gastrointestinal[Title/Abstract])) OR (Gastrointestinal Microbial Community[Title/Abstract])) OR (Gastrointestinal Microbial Communities[Title/Abstract])) OR (Microbial Community, Gastrointestinal[Title/Abstract])) OR (Gastrointestinal Microflora[Title/Abstract])) OR (Microflora, Gastrointestinal[Title/Abstract])) OR (Gastric Microbiome[Title/Abstract])) OR (Gastric Microbiomes[Title/Abstract])) OR (Microbiome, Gastric[Title/Abstract])) OR (Intestinal Microbiome[Title/Abstract])) OR (Intestinal Microbiomes[Title/Abstract])) OR (Microbiome, Intestinal[Title/Abstract])) OR (Intestinal Microbiota[Title/Abstract])) OR (Intestinal Microbiotas[Title/Abstract])) OR (Microbiota, Intestinal[Title/Abstract])) OR (Intestinal Microflora[Title/Abstract])) OR (Microflora, Intestinal[Title/Abstract])) OR (Intestinal Flora[Title/Abstract])) OR (Flora, Intestinal[Title/Abstract])) OR (Enteric Bacteria[Title/Abstract])) OR (Bacteria, Enteric[Title/Abstract]))))) AND ((("Diet"[Mesh]) OR (Diets[Title/Abstract])) OR (dietary[Title/Abstract]) OR nutrition[Title/Abstract] OR ((((((("Life Style"[Mesh]) OR (Life Styles[Title/Abstract])) OR (Lifestyle[Title/Abstract])) OR (Lifestyles[Title/Abstract])) OR (Life Style Induced Illness[Title/Abstract])) OR (Lifestyle Factors[Title/Abstract])) OR (Factor, Lifestyle[Title/Abstract])) OR (Lifestyle Factor[Title/Abstract]) OR ("Physical Fitness"[Mesh]) OR (Fitness, Physical[Title/Abstract]) OR ((((((((((((((((((((((((("Exercise"[Mesh]) OR (Exercises[Title/Abstract])) OR (Physical Activity[Title/Abstract])) OR (Activities, Physical[Title/Abstract])) OR (Activity, Physical[Title/Abstract])) OR (Physical Activities[Title/Abstract])) OR (Exercise, Physical[Title/Abstract])) OR (Exercises, Physical[Title/Abstract])) OR (Physical Exercise[Title/Abstract])) OR (Physical Exercises[Title/Abstract])) OR (Acute Exercise[Title/Abstract])) OR (Acute Exercises[Title/Abstract])) OR (Exercise, Acute[Title/Abstract])) OR (Exercises, Acute[Title/Abstract])) OR (Exercise, Isometric[Title/Abstract])) OR (Exercises, Isometric[Title/Abstract])) OR (Isometric Exercises[Title/Abstract])) OR (Isometric Exercise[Title/Abstract])) OR (Exercise, Aerobic[Title/Abstract])) OR (Aerobic Exercise[Title/Abstract])) OR (Aerobic Exercises[Title/Abstract])) OR (Exercises, Aerobic[Title/Abstract])) OR (Exercise Training[Title/Abstract])) OR (Exercise Trainings[Title/Abstract])) OR (Training, Exercise[Title/Abstract])) OR (Trainings, Exercise[Title/Abstract]) OR (((Activities, Motor[Title/Abstract]) OR (Activity, Motor[Title/Abstract])) OR (Motor Activities[Title/Abstract])) OR ("Motor Activity"[Mesh]))) OR (("Probiotics"[Mesh]) OR (("Probiotics"[Mesh]) OR (Probiotic[Title/Abstract])))) AND ((((((("Cohort Studies"[Mesh]) OR (((((((((((((((((((((((((((((((((Cohort studies[Title/Abstract]) OR (Studies, Cohort[Title/Abstract])) OR (Study, Cohort[Title/Abstract])) OR (Concurrent Studies[Title/Abstract])) OR (Studies, Concurrent[Title/Abstract])) OR (Concurrent Study[Title/Abstract])) OR (Study, Concurrent[Title/Abstract])) OR (Closed Cohort Studies[Title/Abstract])) OR (Cohort Studies, Closed[Title/Abstract])) OR (Closed Cohort Study[Title/Abstract])) OR (Cohort Study, Closed[Title/Abstract])) OR (Study, Closed Cohort[Title/Abstract])) OR (Studies, Closed Cohort[Title/Abstract])) OR (Birth Cohort Studies[Title/Abstract])) OR (Birth Cohort Study[Title/Abstract])) OR (Cohort Studies, Birth[Title/Abstract])) OR (Cohort Study, Birth[Title/Abstract])) OR (Studies, Birth Cohort[Title/Abstract])) OR (Study, Birth Cohort[Title/Abstract])) OR (Analysis, Cohort[Title/Abstract])) OR (Analyses, Cohort[Title/Abstract])) OR (Cohort Analyses[Title/Abstract])) OR (Cohort Analysis[Title/Abstract])) OR (Historical Cohort Studies[Title/Abstract])) OR (Cohort Studies, Historical[Title/Abstract])) OR (Cohort Study, Historical[Title/Abstract])) OR (Historical Cohort Study[Title/Abstract])) OR (Study, Historical Cohort[Title/Abstract])) OR (Studies, Historical Cohort[Title/Abstract])) OR (Incidence Studies[Title/Abstract])) OR (Incidence Study[Title/Abstract])) OR (Studies, Incidence[Title/Abstract])) OR (Study, Incidence[Title/Abstract]))) OR ("Case Reports" [Publication Type]) OR (((Case Study[Title/Abstract]) OR (Case Studies[Title/Abstract])) OR (Case Histories[Title/Abstract]))) OR (Prospective cohort[Title/Abstract])) OR (case controls[Title/Abstract])) OR (case-controls[Title/Abstract])) OR (case control[Title/Abstract])) | 1331 |
| #8 | #1 OR #2 OR #3 OR #4 OR #5 OR #6 OR #7 OR #8 | 155 |

- 1. **Cochrane search stratergy for 48**

| **Sequence** | **Search words** | **Hits** |
| --- | --- | --- |
| #1 | (pregnancy diabetes mellitus ):ti,ab,kw OR (Pregnancy in Diabetic):ti,ab,kw OR (Pregnancy in Diabetes):ti,ab,kw OR (Pregnancy in Diabete):ti,ab,kw OR (Pregnancy):ti,ab,kw OR (Pregnent):ti,ab,kw | 64861 |
| #2 | (alkaline diet):ti,ab,kw OR (artificial diet):ti,ab,kw OR (Atkins diet):ti,ab,kw OR (carbohydrate diet):ti,ab,kw OR (carbohydrate loading diet):ti,ab,kw OR (cariogenic diet):ti,ab,kw OR (casein free diet):ti,ab,kw OR (cereal-based diet):ti,ab,kw OR (DASH diet):ti,ab,kw OR (elemental diet):ti,ab,kw OR (elimination diet):ti,ab,kw OR (experimental diet):ti,ab,kw OR (fad diet):ti,ab,kw OR (fiber free diet):ti,ab,kw OR (gluten free diet):ti,ab,kw OR (healthy diet):ti,ab,kw OR (high calorie diet):ti,ab,kw OR (high fiber diet):ti,ab,kw OR (high glycemic index diet):ti,ab,kw OR (high salt diet):ti,ab,kw OR (intuitive eating):ti,ab,kw OR (ketogenic diet):ti,ab,kw OR (lactose free diet):ti,ab,kw OR (lipid diet):ti,ab,kw OR (liquid diet):ti,ab,kw OR (low calorie diet):ti,ab,kw OR (low carbohydrate diet):ti,ab,kw OR (low fiber diet):ti,ab,kw OR (low FODMAP diet):ti,ab,kw OR (low glycemic index diet):ti,ab,kw OR (low iodine diet):ti,ab,kw OR (low residue diet):ti,ab,kw OR (macrobiotic diet):ti,ab,kw OR (Mediterranean diet):ti,ab,kw OR (Nordic diet):ti,ab,kw OR (obesogenic diet):ti,ab,kw OR (Okinawan diet):ti,ab,kw OR (paleolithic diet):ti,ab,kw OR (protein diet):ti,ab,kw OR (raw food diet):ti,ab,kw OR (soft diet):ti,ab,kw OR (unhealthy diet):ti,ab,kw OR (vegetarian diet):ti,ab,kw OR (Western diet):ti,ab,kw OR (diet):ti,ab,kw OR (dietary):ti,ab,kw | 96901 |
| #3 | (nutrition):ti,ab,kw OR (adolescent nutrition):ti,ab,kw OR (carbon source):ti,ab,kw OR (child nutrition):ti,ab,kw OR (dietary intake):ti,ab,kw OR (dietary pattern):ti,ab,kw OR (fat content):ti,ab,kw OR (fat load):ti,ab,kw OR (feeding behavior):ti,ab,kw OR (food access):ti,ab,kw OR (food availability):ti,ab,kw OR (food chain):ti,ab,kw OR (food frequency questionnaire):ti,ab,kw OR (food intake):ti,ab,kw OR (geriatric nutrition):ti,ab,kw OR (glycemic index):ti,ab,kw OR (glycemic load):ti,ab,kw OR (immunonutrition):ti,ab,kw OR (maternal nutrition):ti,ab,kw OR (nutrient availability):ti,ab,kw OR (nutrient concentration):ti,ab,kw OR (nutrient content):ti,ab,kw OR (nutrient cycle):ti,ab,kw OR (nutrient cycling):ti,ab,kw OR (nutrient dynamics):ti,ab,kw OR (nutrient limitation):ti,ab,kw OR (nutrient loading):ti,ab,kw OR (nutrient management):ti,ab,kw OR (nutrient supply):ti,ab,kw OR (nutrient uptake):ti,ab,kw OR (nutrition education):ti,ab,kw OR (nutritional assessment):ti,ab,kw OR (nutrition labeling):ti,ab,kw OR (nutritional counseling):ti,ab,kw OR (nutritional health):ti,ab,kw OR (nutritional requirement):ti,ab,kw OR (nutritional status):ti,ab,kw OR (nutritional tolerance):ti,ab,kw OR (nutritional value):ti,ab,kw OR (personalized nutrition):ti,ab,kw OR (plant nutrition):ti,ab,kw OR (sport nutrition):ti,ab,kw OR (water deprivation):ti,ab,kw | 80862 |
| #4 | (Life Style):ti,ab,kw OR (Life Styles):ti,ab,kw OR (Lifestyle):ti,ab,kw OR (Lifestyles):ti,ab,kw OR (Life Style Induced Illness):ti,ab,kw OR (Lifestyle Factors):ti,ab,kw OR (Factor, Lifestyle):ti,ab,kw OR (Lifestyle Factor):ti,ab,kw | 26173 |
| #5 | (Physical Fitness):ti,ab,kw OR (Fitness, Physical):ti,ab,kw | 9475 |
| #6 | (Exercise):ti,ab,kw OR (Exercises):ti,ab,kw OR (Physical Activity):ti,ab,kw OR (Activities, Physical):ti,ab,kw OR (Activity, Physical):ti,ab,kw OR (Physical Activities):ti,ab,kw OR (Exercise, Physical):ti,ab,kw OR (Exercises, Physical):ti,ab,kw OR (Physical Exercise):ti,ab,kw OR (Physical Exercises):ti,ab,kw OR (Acute Exercise):ti,ab,kw OR (Acute Exercises):ti,ab,kw OR (Exercise, Acute):ti,ab,kw OR (Exercises, Acute):ti,ab,kw OR (Exercise, Isometric):ti,ab,kw OR (Exercises, Isometric):ti,ab,kw OR (Isometric Exercises):ti,ab,kw OR (Isometric Exercise):ti,ab,kw OR (Exercise, Aerobic):ti,ab,kw OR (Aerobic Exercises):ti,ab,kw OR (Aerobic Exercise):ti,ab,kw OR (Exercises, Aerobic):ti,ab,kw OR (Exercise Training):ti,ab,kw OR (Exercise Trainings):ti,ab,kw OR (Training, Exercise):ti,ab,kw OR (Trainings, Exercise):ti,ab,kw | 146983 |
| #7 | (Motor Activity):ti,ab,kw OR (Activities, Motor):ti,ab,kw OR (Activity, Motor):ti,ab,kw OR (Motor Activities):ti,ab,kw | 13453 |
| #8 | (probiotic agent):ti,ab,kw OR (Probiotic):ti,ab,kw OR (Probiotics):ti,ab,kw | 8241 |
| #9 | (Cohort Studies):ti,ab,kw OR (Cohort Study):ti,ab,kw OR (Studies, Cohort):ti,ab,kw OR (Study, Cohort):ti,ab,kw OR (Concurrent Studies):ti,ab,kw OR (Studies, Concurrent):ti,ab,kw OR (Concurrent Study):ti,ab,kw OR (Study, Concurrent):ti,ab,kw OR (Closed Cohort Studies):ti,ab,kw OR (Cohort Studies, Closed):ti,ab,kw OR (Closed Cohort Study):ti,ab,kw OR (Cohort Study, Closed):ti,ab,kw OR (Study, Closed Cohort):ti,ab,kw OR (Studies, Closed Cohort):ti,ab,kw OR (Birth Cohort Studies):ti,ab,kw OR (Birth Cohort Study):ti,ab,kw OR (Cohort Studies, Birth):ti,ab,kw OR (Cohort Study, Birth):ti,ab,kw OR (Studies, Birth Cohort):ti,ab,kw OR (Study, Birth Cohort):ti,ab,kw OR (Analysis, Cohort):ti,ab,kw OR (Analyses, Cohort):ti,ab,kw OR (Cohort Analyses):ti,ab,kw OR (Cohort Analysis):ti,ab,kw OR (Historical Cohort Studies):ti,ab,kw OR (Cohort Studies, Historical):ti,ab,kw OR (Cohort Study, Historical):ti,ab,kw OR (Historical Cohort Study):ti,ab,kw OR (Study, Historical Cohort):ti,ab,kw OR (Studies, Historical Cohort):ti,ab,kw OR (Incidence Study):ti,ab,kw OR (Incidence Studies):ti,ab,kw OR (Studies, Incidence):ti,ab,kw OR (Case Reports):ti,ab,kw OR (Study, Incidence):ti,ab,kw OR (Cohort Study):ti,ab,kw OR (Studies, Cohort):ti,ab,kw OR (Study, Cohort):ti,ab,kw OR (Concurrent Studies):ti,ab,kw OR (Studies, Concurrent):ti,ab,kw OR (Concurrent Study):ti,ab,kw OR (Study, Concurrent):ti,ab,kw OR (Closed Cohort Studies):ti,ab,kw OR (Cohort Studies, Closed):ti,ab,kw OR (Closed Cohort Study):ti,ab,kw OR (Cohort Study, Closed):ti,ab,kw OR (Study, Closed Cohort):ti,ab,kw OR (Studies, Closed Cohort):ti,ab,kw OR (Birth Cohort Studies):ti,ab,kw OR (Birth Cohort Study):ti,ab,kw OR (Cohort Studies, Birth):ti,ab,kw OR (Cohort Study, Birth):ti,ab,kw OR (Studies, Birth Cohort):ti,ab,kw OR (Study, Birth Cohort):ti,ab,kw OR (Analysis, Cohort):ti,ab,kw OR (Analyses, Cohort):ti,ab,kw OR (Cohort Analyses):ti,ab,kw OR (Cohort Analysis):ti,ab,kw OR (Historical Cohort Studies):ti,ab,kw OR (Cohort Studies, Historical):ti,ab,kw OR (Cohort Study, Historical):ti,ab,kw OR (Historical Cohort Study):ti,ab,kw OR (Study, Historical Cohort):ti,ab,kw OR (Studies, Historical Cohort):ti,ab,kw OR (Incidence Studies):ti,ab,kw OR (Incidence Study):ti,ab,kw OR (Study, Incidence):ti,ab,kw OR (Studies, Incidence):ti,ab,kw OR (Prospective cohort):ti,ab,kw OR (case controls):ti,ab,kw OR (case-controls):ti,ab,kw OR (case control):ti,ab,kw | 203380 |
| #10 | (16sRNA):ti,ab,kw OR (metagenomics):ti,ab,kw OR (Community Genomics):ti,ab,kw OR (Genomics, Community):ti,ab,kw OR (Population Genomics):ti,ab,kw OR (Genomics, Population):ti,ab,kw | 512 |
| #11 | (Gastrointestinal Microbiome):ti,ab,kw OR (Microbiome, Gastrointestinal):ti,ab,kw OR (Gastrointestinal Microbiomes):ti,ab,kw OR (Gut Microbiome):ti,ab,kw OR (Gut Microbiomes):ti,ab,kw OR (Microbiome, Gut):ti,ab,kw OR (Gut Microflora):ti,ab,kw OR (Microflora, Gut):ti,ab,kw OR (Gut Microbiota):ti,ab,kw OR (Gut Microbiotas):ti,ab,kw OR (Gastrointestinal Flora):ti,ab,kw OR (Microbiota, Gut):ti,ab,kw OR (Flora, Gastrointestinal):ti,ab,kw OR (Gut Flora):ti,ab,kw OR (Gastrointestinal Microbiota):ti,ab,kw OR (Flora, Gut):ti,ab,kw OR (Gastrointestinal Microbiotas):ti,ab,kw OR (Microbiota, Gastrointestinal):ti,ab,kw OR (Gastrointestinal Microbial Community):ti,ab,kw OR (Gastrointestinal Microbial Communities):ti,ab,kw OR (Microbial Community, Gastrointestinal):ti,ab,kw OR (Gastrointestinal Microflora):ti,ab,kw OR (Microflora, Gastrointestinal):ti,ab,kw OR (Gastric Microbiome):ti,ab,kw OR (Gastric Microbiomes):ti,ab,kw OR (Intestinal Microbiome):ti,ab,kw OR (Microbiome, Gastric):ti,ab,kw OR (Intestinal Microbiomes):ti,ab,kw OR (Microbiome, Intestinal):ti,ab,kw OR (Intestinal Microbiota):ti,ab,kw OR (Intestinal Microbiotas):ti,ab,kw OR (Microbiota, Intestinal):ti,ab,kw OR (Intestinal Microflora):ti,ab,kw OR (Microflora, Intestinal):ti,ab,kw OR (Intestinal Flora):ti,ab,kw OR (Flora, Intestinal):ti,ab,kw OR (Enteric Bacteria):ti,ab,kw OR (Bacteria, Enteric):ti,ab,kw | 7501 |
| #12 | #2 OR #3 OR #4 OR #5 OR #6 OR #7 OR #8 | 273063 |
| #13 | #10 OR #11 | 7742 |
| #14 | #1 AND #9 AND #12 AND #13 | 53 |
| #15 | "#14 - #1 AND #9 AND #12 AND #13" with Cochrane Library publication date Between Jul 2012 and Jul 2022 | 48 |

- 1. **Web of Science search stratergy for 395**

| **Sequence** | **Search words** | **Hits** |
| --- | --- | --- |
| #1 | (((((TS=(pregnancy diabetes mellitus )) OR TS=(Pregnancy in Diabetic)) OR TS=(Pregnancy in Diabetes)) OR TS=(Pregnancy in Diabete)) OR TS=(Pregnancy)) OR TS=(Pregnent) | 1348829 |
| #2 | (((((((((((((((((((((((((((((((((((((((((((((((((((((((((((((((((((((((((((((((((((((((((((((((((((((((((((((((((((((((((((((((((((TS=(alkaline diet)) OR TS=(artificial diet)) OR TS=(Atkins diet)) OR TS=(carbohydrate diet)) OR TS=(carbohydrate loading diet)) OR TS=(cariogenic diet)) OR TS=(casein free diet)) OR TS=(cereal-based diet)) OR TS=(DASH diet)) OR TS=(elemental diet)) OR TS=(elimination diet)) OR TS=(experimental diet)) OR TS=(fad diet)) OR TS=(fiber free diet)) OR TS=(gluten free diet)) OR TS=(healthy diet)) OR TS=(high calorie diet)) OR TS=(high fiber diet)) OR TS=(high glycemic index diet)) OR TS=(high salt diet)) OR TS=(intuitive eating)) OR TS=(ketogenic diet)) OR TS=(lactose free diet)) OR TS=(lipid diet)) OR TS=(liquid diet)) OR TS=(low calorie diet)) OR TS=(low carbohydrate diet)) OR TS=(low fiber diet)) OR TS=(low FODMAP diet)) OR TS=(low glycemic index diet)) OR TS=(low iodine diet)) OR TS=(low residue diet)) OR TS=(macrobiotic diet)) OR TS=(Mediterranean diet)) OR TS=(Nordic diet)) OR TS=(obesogenic diet)) OR TS=(Okinawan diet)) OR TS=(paleolithic diet)) OR TS=(protein diet)) OR TS=(raw food diet)) OR TS=(soft diet)) OR TS=(unhealthy diet)) OR TS=(vegetarian diet)) OR TS=(Western diet)) OR TS=(diet)) OR TS=(dietary)) OR TS=(nutrition)) OR TS=(adolescent nutrition)) OR TS=(carbon source)) OR TS=(child nutrition)) OR TS=(dietary intake)) OR TS=(dietary pattern)) OR TS=(fat content)) OR TS=(fat load)) OR TS=(feeding behavior)) OR TS=(food access)) OR TS=(food availability)) OR TS=(food chain)) OR TS=(food frequency questionnaire)) OR TS=(food intake)) OR TS=(geriatric nutrition)) OR TS=(glycemic index)) OR TS=(glycemic load)) OR TS=(immunonutrition)) OR TS=(maternal nutrition)) OR TS=(nutrient availability)) OR TS=(nutrient concentration)) OR TS=(nutrient content)) OR TS=(nutrient cycle)) OR TS=(nutrient cycling)) OR TS=(nutrient dynamics)) OR TS=(nutrient dynamics)) OR TS=(nutrient loading)) OR TS=(nutrient management)) OR TS=(nutrient supply)) OR TS=(nutrient uptake)) OR TS=(nutrition education)) OR TS=(nutrition labeling)) OR TS=(nutritional assessment)) OR TS=(nutritional counseling)) OR TS=(nutritional health)) OR TS=(nutritional requirement)) OR TS=(nutritional status)) OR TS=(nutritional tolerance)) OR TS=(nutritional value)) OR TS=(personalized nutrition)) OR TS=(plant nutrition)) OR TS=(sport nutrition)) OR TS=(water deprivation)) OR TS=(Life Style)) OR TS=(Life Styles)) OR TS=(Lifestyle)) OR TS=(Lifestyles)) OR TS=(Life Style Induced Illness)) OR TS=(Lifestyle Factors)) OR TS=(Factor, Lifestyle)) OR TS=(Lifestyle Factor)) OR TS=(Physical Fitness)) OR TS=(Fitness, Physical)) OR TS=(Exercise)) OR TS=(Exercises)) OR TS=(Physical Activity)) OR TS=(Activities, Physical)) OR TS=(Activity, Physical)) OR TS=(Physical Activities)) OR TS=(Exercise, Physical)) OR TS=(Exercises, Physical)) OR TS=(Physical Exercise)) OR TS=(Physical Exercises)) OR TS=(Acute Exercise)) OR TS=(Acute Exercises)) OR TS=(Exercise, Acute)) OR TS=(Exercises, Acute)) OR TS=(Exercise, Isometric)) OR TS=(Exercises, Isometric)) OR TS=(Isometric Exercises)) OR TS=(Isometric Exercise)) OR TS=(Exercise, Aerobic)) OR TS=(Aerobic Exercise)) OR TS=(Aerobic Exercises)) OR TS=(Exercises, Aerobic)) OR TS=(Exercise Training)) OR TS=(Exercise Trainings)) OR TS=(Training, Exercise)) OR TS=(Trainings, Exercise)) OR TS=(Motor Activity)) OR TS=(Activities, Motor)) OR TS=(Activity, Motor)) OR TS=(Motor Activities)) OR TS=(probiotic agent)) OR TS=(Probiotic)) OR TS=(Probiotics) | 6729484 |
| #3 | ((((((((((((((((((((((((((((((((((((((((((((((((((((((((((((((((((((((((TS=(Cohort Studies)) OR TS=(Cohort Study)) OR TS=(Studies, Cohort)) OR TS=(Study, Cohort)) OR TS=(Concurrent Studies)) OR TS=(Studies, Concurrent)) OR TS=(Studies, Concurrent)) OR TS=(Concurrent Study)) OR TS=(Study, Concurrent)) OR TS=(Closed Cohort Studies)) OR TS=(Cohort Studies, Closed)) OR TS=(Closed Cohort Study)) OR TS=(Cohort Study, Closed)) OR TS=(Study, Closed Cohort)) OR TS=(Studies, Closed Cohort)) OR TS=(Birth Cohort Studies)) OR TS=(Birth Cohort Study)) OR TS=(Cohort Studies, Birth)) OR TS=(Cohort Study, Birth)) OR TS=(Studies, Birth Cohort)) OR TS=(Study, Birth Cohort)) OR TS=(Analysis, Cohort)) OR TS=(Analyses, Cohort)) OR TS=(Cohort Analyses)) OR TS=(Cohort Analysis)) OR TS=(Historical Cohort Studies)) OR TS=(Cohort Studies, Historical)) OR TS=(Cohort Study, Historical)) OR TS=(Historical Cohort Study)) OR TS=(Study, Historical Cohort)) OR TS=(Studies, Historical Cohort)) OR TS=(Incidence Studies)) OR TS=(Incidence Study)) OR TS=(Studies, Incidence)) OR TS=(Study, Incidence)) OR TS=(Case Reports)) OR TS=(Cohort Study)) OR TS=(Studies, Cohort)) OR TS=(Study, Cohort)) OR TS=(Concurrent Studies)) OR TS=(Studies, Concurrent)) OR TS=(Concurrent Study)) OR TS=(Study, Concurrent)) OR TS=(Closed Cohort Studies)) OR TS=(Cohort Studies, Closed)) OR TS=(Closed Cohort Study)) OR TS=(Cohort Study, Closed)) OR TS=(Study, Closed Cohort)) OR TS=(Studies, Closed Cohort)) OR TS=(Birth Cohort Studies)) OR TS=(Birth Cohort Study)) OR TS=(Cohort Studies, Birth)) OR TS=(Cohort Study, Birth)) OR TS=(Studies, Birth Cohort)) OR TS=(Study, Birth Cohort)) OR TS=(Analysis, Cohort)) OR TS=(Analyses, Cohort)) OR TS=(Cohort Analyses)) OR TS=(Cohort Analysis)) OR TS=(Historical Cohort Studies)) OR TS=(Cohort Studies, Historical)) OR TS=(Cohort Study, Historical)) OR TS=(Historical Cohort Study)) OR TS=(Study, Historical Cohort)) OR TS=(Studies, Historical Cohort)) OR TS=(Incidence Studies)) OR TS=(Incidence Study)) OR TS=(Studies, Incidence)) OR TS=(Study, Incidence)) OR TS=(Prospective cohort)) OR TS=(case controls)) OR TS=(case-controls)) OR TS=(case control) | 5318487 |
| #4 | ((((((((((((((((((((((((((((((((((((((((((((TS=(16sRNA)) OR TS=(metagenomics)) OR TS=(Community Genomics)) OR TS=(Genomics, Community)) OR TS=(Population Genomics)) OR TS=(Genomics, Population)) OR TS=(Environmental Genomics)) OR TS=(Gastrointestinal Microbiome)) OR TS=(Gastrointestinal Microbiomes)) OR TS=(Microbiome, Gastrointestinal)) OR TS=(Gut Microbiome)) OR TS=(Gut Microbiomes)) OR TS=(Microbiome, Gut)) OR TS=(Gut Microflora)) OR TS=(Microflora, Gut)) OR TS=(Gut Microbiota)) OR TS=(Gut Microbiotas)) OR TS=(Microbiota, Gut)) OR TS=(Gastrointestinal Flora)) OR TS=(Flora, Gastrointestinal)) OR TS=(Gut Flora)) OR TS=(Flora, Gut)) OR TS=(Gastrointestinal Microbiota)) OR TS=(Gastrointestinal Microbiotas)) OR TS=(Microbiota, Gastrointestinal)) OR TS=(Gastrointestinal Microbial Community)) OR TS=(Gastrointestinal Microbial Communities)) OR TS=(Microbial Community, Gastrointestinal)) OR TS=(Gastrointestinal Microflora)) OR TS=(Microflora, Gastrointestinal)) OR TS=(Gastric Microbiome)) OR TS=(Gastric Microbiomes)) OR TS=(Microbiome, Gastric)) OR TS=(Intestinal Microbiome)) OR TS=(Intestinal Microbiomes)) OR TS=(Microbiome, Intestinal)) OR TS=(Intestinal Microbiota)) OR TS=(Intestinal Microbiotas)) OR TS=(Microbiota, Intestinal)) OR TS=(Intestinal Microflora)) OR TS=(Microflora, Intestinal)) OR TS=(Intestinal Flora)) OR TS=(Flora, Intestinal)) OR TS=(Enteric Bacteria)) OR TS=(Bacteria, Enteric) | 284045 |
| #5 | #1 AND #2 AND #3 AND #4 | 470 |
| #6 | #1 AND #2 AND #3 AND #4 and English (Languages) | 466 |
| #7 | #1 AND #2 AND #3 AND #4 and English (Languages) year 2012-07-05-2022-07-05 | 395 |

**Supplementary Table 2.** **Quality assessment of the included studies using the Newcastle-Ottawa Scale.**

| **Study** | **Selection** | **Comparability** | **Exposure** | **Total** |
| --- | --- | --- | --- | --- |
| Bahiyah Abdullah,2022 | 4 | 1 | 3 | 8 |
| Ting Chen,2021 | 4 | 2 | 2 | 8 |
| Thomas P. Mullins,2021 | 4 | 2 | 2 | 8 |
| Kei TANAKA,2022 | 4 | 1 | 3 | 8 |
| J. Wei,2021 | 4 | 1 | 3 | 8 |
| J. S. Gámez-Valdez，2021 | 4 | 2 | 2 | 8 |
| Wei Zheng,2020 | 4 | 0 | 3 | 7 |
| Ping Hu,2021 | 4 | 1 | 2 | 7 |
| Yao Su,2021a | 4 | 1 | 2 | 7 |
| Mengjun Cu,2019 | 4 | 0 | 3 | 7 |
| Lingling Huang,2021 | 4 | 1 | 2 | 7 |
| Shujuan Ma,2020 | 4 | 0 | 3 | 7 |
| Palin Sililas,2021 | 4 | 0 | 2 | 6 |
| Omry Koren,2021 | 4 | 1 | 1 | 6 |
| Genxia Li,2021 | 3 | 0 | 3 | 6 |
| Ya-Shu kuang,2017 | 4 | 0 | 2 | 6 |
| Xing Wang,2020 | 4 | 0 | 2 | 6 |
| Yanxin Wu,2019 | 4 | 0 | 2 | 6 |
| Yajuan Xu,2020 | 4 | 0 | 2 | 6 |
| Guangyong Ye，2019 | 4 | 1 | 1 | 6 |
| Yao Su,2021b | 4 | 0 | 1 | 5 |
| Haowen Zhang,2021 | 4 | 0 | 1 | 5 |

**Supplementary Table 3. Study characteristics of each study focusing on basic data.**

| **Study** | **Country** | **Publication year** | **Study type** | **Age** | | **Pre-BMI** | |
| --- | --- | --- | --- | --- | --- | --- | --- |
|  |  |  |  | **GDM** | **NGDM** | **GDM** | **NGDM** |
| Bahiyah Abdullah,2022 | malaysia | 2022 | Cohort | 30.42±4.03 | 30.62±3.80 | 27.37±4.61 | 24.41±5.75 |
| Ting Chen,2021 | China | 2021 | Case study |  | 30-35 | 21.4±3.2 | 21.4±3.1 |
| Mengjun Cu,2019 | China | 2019 | Case study | 29.00 (27.00–31.00) | 28.00 (25.00–33.00) | 21.77 (19.47–23.84)/ | 21.60 (19.69–24.46) |
| Ping Hu,2021 | China | 2021 | Nested case control | 28.2±4.4 | 27.9±4.0 | 21.9±3.2 | 21.2±3.4 |
| Omry Koren,2021 | America | 2021 | Cohort study | NA | NA | NA | NA |
| Genxia Li,2021 | China | 2021 | case control | 29.80 ± 2.19 | 29.00 ± 1.88 | 23.64 ± 1.36 | 21.39 ± 1.37 |
| Palin Sililas,2021 | Thailand | 2021 | case control | 32.6 ± 4.6 | 30.9 ± 5.5 | 24.5 ± 5.0 | 22.9 ± 4.5 |
| Yao Su,2021 | China | 2021 | case control | 30.5 ± 3.96 | 30.6 ± 4.47 |  |  |
| Yao Su,2021 | China | 2021 | Cohort study | 28.7±3.42 | 31.5±4.56 | 21.7 (19.96–23.47) | 22.6 (18.95–25.25) |
| Kei TANAKA,2022 | Japan | 2022 | Cohort study | 38 (29–45) | 40 (24–43) | 24.3 (17.8–34.4) | 21.3 (16.2–37.2) |
| Xing Wang,2020 | China | 2020 | case control | 30.56 ± 4.24 | 29.19 ± 3.04 | 21.45 ± 2.92 | 20.46 ± 2.61 |
| Yanxin Wu,2019 | China | 2019 | Cohort study | 36 (32–38.5) | 32.5 (30–35) | 22.58 (19.42–25.58)/ | 20.96 (19.70–22.17) |
| Yajuan Xu,2020 | China | 2020 | case control | 33.7±4.7 | 32.3±4.3 | 24±3.6 | 22±3.1 |
| Wei Zheng,2020 | China | 2020 | Cohort study | 32.58±4.1 | 31.79±3.70 | 22.57±2.85 | 21.32±3.00 |
| J. Wei,2021 | China | 2021 | Cohort study | 30.1±3.5 | 26.1±3.6 | 24.7±4.1 | 21.1±2.3 |
| Shujuan Ma,2020 | China | 2020 | Nested case control | 31.0 (28.8–35.0) | 31.5 (28.75–35) | 22.79 ± 3.01 | 20.83 ± 2.65 |
| Thomas P. Mullins,2021 | Austria | 2021 | Cohort study | 34.2±0.9 | 33.3±1.0 | 33.7±0.9 | 33.9±1.0 |
| Haowen Zhang,2021 | China | 2021 | case control | 34.50 (31.75–38.00) | 38.00 (36.25–39.00) | 38.00 (36.25–39.00)/ | 20.75 (19.29–24.08) |
| Guangyong Ye,2019 | China | 2019 | case control | 34.3 ± 3.8 | 30.8 ± 4.8 | NA | NA |
| J. S. Gámez-Valdez,2021 | Mexico | 2021 | case control | NA | NA | NA | NA |
| Lingling Huang,2021 | Thailand | 2021 | case control | 31.16 ± 0.86 | 29.04±1.09 | 22.99 ± 0.73 | 24.05±1.05 |
| Ya-Shu kuang,2017 | China | 2017 | case control | 30.5±3.3 | 28.8±3.1 | 21.9±3.1 | 20.2±2.0 |

**N/A, not available.**

**Supplementary Table 4. Study characteristics of each study focusing on stool sample storage, extraction and analysis.**

| **Study** | **Diagnose** | **Sample Storage** | **DNA Extraction** | **Target** | **SequencingTechniqu** | **Clustering** | **Database** |
| --- | --- | --- | --- | --- | --- | --- | --- |
| Bahiyah Abdullah,2022 | OGTT | -20 | Stool DNA Isolation Kit (NORGEN, Canada) | 16sRNAV3-V4 | Illumina MiSeq  platform (Illumina, USA) | 99%OTUs | Silva |
| Ting Chen,2021 | OGTT | -80 | QIAGEN, Hilden, Germany | 16sRNAV3-V4 | MiSeq platform | 99% OTUs | Greengenes |
| Mengjun Cu,2019 | OGTT | −80°C | Ezup column bacterial genomic DNA  extraction kit | 16S rRNA V4 | Illumina Miseq 2 × 300 platform in BGI-Wuhan | 97%OTUs | Greengenes |
| Ping Hu,2021 | OGTT | NA | NA | 16S rRNA V4 | Illumina MiSeq platform (Illumina, USA) | NA | NA |
| Omry Koren,2021 | OGTT | NA | NA | 16sRNA V1-V2 | Illumina HiSeq 2000 | 97%OTUs | NA |
| Genxia Li,2021 | OGTT | −80°C | BGI Stool  Genome Extraction Kit | 16SrRNA V3-V4 | Illumina Hiseq 2500 PE250 | 97%OTUs | Greengenes |
| Palin Sililas,2021 | NDDG | −80°C | genomic  DNA isolation kit | NA | NA | NA | NA |
| Yao Su,2021a | OGTT | −80°C | Qubit dsDNA HS Assay Kit | 16SrRNA V3-V4 | Illumina Hiseq 2500 platform | 97%OTUs | SILVA |
| Yao Su,2021b | OGTT | −80°C | Qiagen QIAamp DNA Stool  Mini Kits | 16SrRNA V3-V4 | Illumina MiSeq instrument | 97%OTUs | SILVA |
| Kei TANAKA,2022 | OGTT | −80°C | NucleoSpin® DNA Stool kit | 16SrRNA V3-V4 | QIIME2 platform ( | 99%OTUs | SILVA |
| Xing Wang,2020 | OGTT | −80°C | e OMEGA-soil DNA Kit | 16SrRNA V3-V4 | Illumina MiSeq | 97%OTUs | NA |
| Yanxin Wu,2019 | OGTT | −80°C | MoBio PowerFecal DNA  Kit | NA | IlluminaHiSeq2500 | 95%OTUs | NA |
| Yajuan Xu,2020 | OGTT | −80°C | NA | 16SrRNA V3-V4 | Illumina Hiseq 2500 PE250 | 99%OTUs | NA |
| Wei Zheng,2020 | OGTT | −80°C | PSP Spin Stool DNA Plus kit | 16SrRNA V3-V4 | Illumina Hiseq 2500 PE250 | 97%OTUs | RDP database |
| J. Wei,2021 | OGTT | −80℃ | MagPure  Stool DNA KF kit B | 16S rRNA V4 | Illumina HiSeq 2500 platform | 97%OTUs | Greengenes |
| Shujuan Ma,2020 | OGTT | −20◦C | QIAamp Fast  DNA Stool Mini Kit | 16S rRNA V4 | QIIME2 | 100%OTUs | SILVA |
| Thomas P. Mullins,2021 | OGTT | −80℃ | QIAGEN AllPrep DNA extraction kit | 16S rRNAV6-V8 | Illumina MiSeq platform | 97%OTUs | Greengenes |
| Haowen Zhang,2021 | OGTT | −80℃ | NA | 16SrRNA V3-V4 | NA | NA | NA |
| Guangyong Ye,2019 | OGTT | −80℃ | QIAamp DNA Stool Mini Kit (Qiagen, Hilden, Germany) | 16SrRNA V3-V4 | Illumina MiSeq platform | 97%OTUs | RDP database |
| J. S. Gámez-Valdez,2021 | OGTT | -20 °C | NA | 16SrRNA V3-V4 | Illumina MiSeq (2 × 300) | 99%OTUs | Silva |
| Lingling Huang,2021 | NA | NA | NA | NA | NA | NA | NA |
| Ya-Shu kuang,2017 | OGTT | –20°C | NA | metagenomic sequences | IDBA-UD v. 1.1.1 | NA | NA |

**N/A, not available.**

**Supplementary Table 5. The relationships between intestinal microbiota and biochemical indicators.**

| **Pylma** | **Family** | **Genus** | **MGTT-1H** | **MGTT-2H** | **insulin** | **FBG** | **HbAlc** | **LDH** | **HDL** | **Study** |
| --- | --- | --- | --- | --- | --- | --- | --- | --- | --- | --- |
| Bacteroidetes | Bacteroidaceae | Bacteroides | ↓ |  |  |  |  |  | ↑ | Chen el ta (14)，Su el ta (23) |
|  |  | Parabacteroides |  |  |  | ↓ |  |  |  | Chen el ta (14). |
| Firmicutes | Ruminococcaceae | Faecalibacterium | ↓ | ↓ |  |  | ↓ |  | ↑ | Chen el ta (14)，Su el ta (23),Ye el ta (33) |
|  |  | Oscillospira | ↓ |  |  |  |  |  |  | Chen el ta (14) |
|  | Lachnospiraceae | Ruminococcus | ↓ | ↓ |  | ↑ | ↓ |  | ↑ | Koren (42)，Su el ta (23)，Wang el ta (75) |
|  |  | lachnospiraceae | ↑ |  |  |  |  |  |  | Wang el ta (75) |
|  |  | Blautia |  |  |  | ↑ |  |  |  | Ye el ta (33) |
| Proteobacteria | Enterobacteriaceae | Shigella |  |  | ↑ |  |  |  |  | Cui el ta (15) |
|  |  | - |  |  |  | ↓ |  |  |  | Chen el ta (14) |
| Verrucomicrobia | Verrucomicrobiaceae | Akkermansia | ↓ |  |  |  |  |  |  | Su el ta (24) |


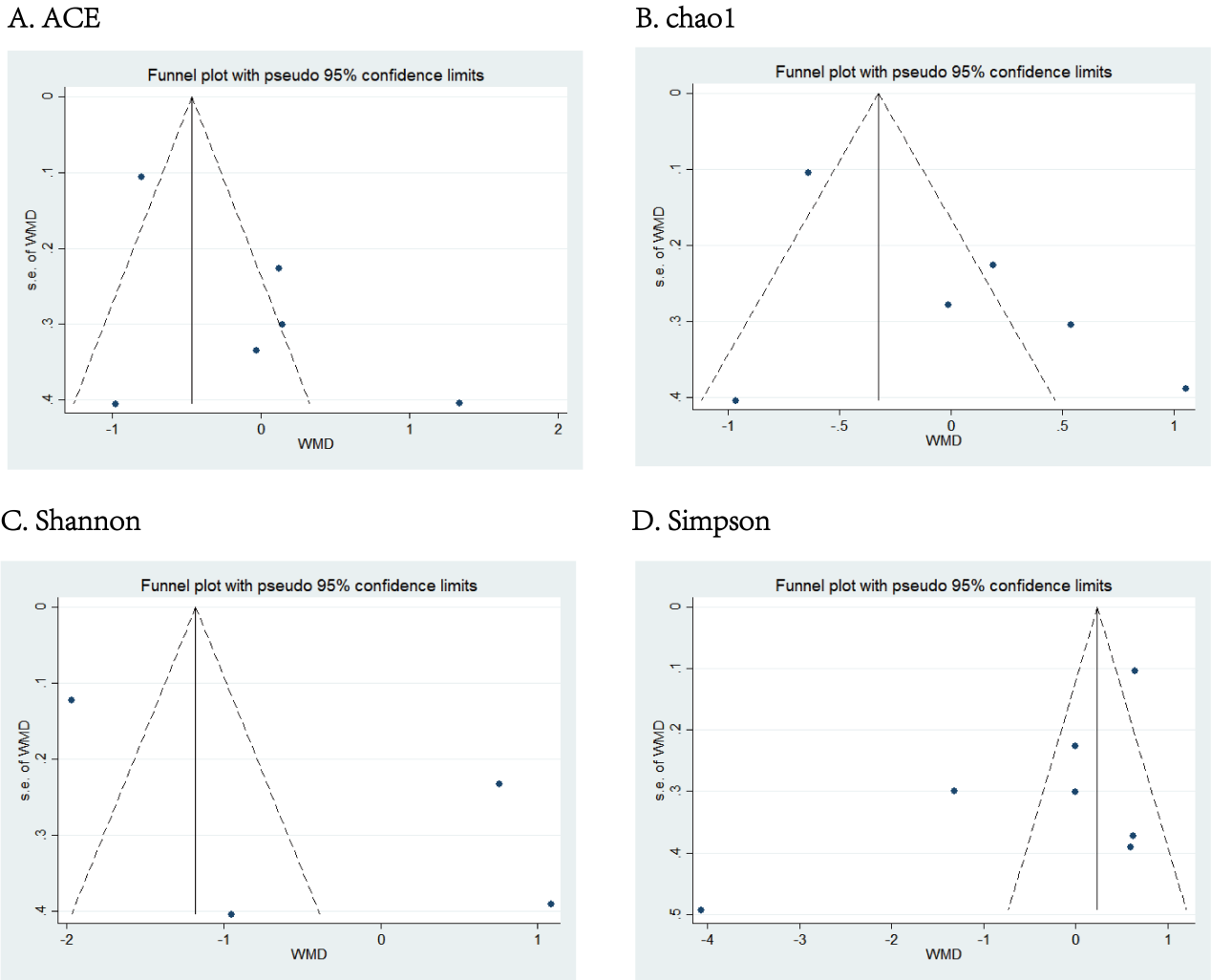


**Supplementary Figure 1. Funnel plots assessing publication bias. A. ACE, B. Chao1, C. Shannon, D. Simpson**
